# New Specimens of the Rare Taeniodont *Wortmania* (Mammalia: Eutheria) from the San Juan Basin of New Mexico and Comments on the Phylogeny and Functional Morphology of “Archaic” Mammals

**DOI:** 10.1371/journal.pone.0075886

**Published:** 2013-09-30

**Authors:** Thomas E. Williamson, Stephen L. Brusatte

**Affiliations:** 1 New Mexico Museum of Natural History and Science, Department of Research and Collections, Albuquerque, New Mexico, United States of America; 2 School of GeoSciences, University of Edinburgh, Edinburgh, Scotland, United Kingdom; 3 Division of Paleontology, American Museum of Natural History, New York, New York, United States of America; 4 Department of Earth and Environmental Sciences, Columbia University, Palisades, New York, United States of America; Team 'Evo-Devo of Vertebrate Dentition', France

## Abstract

**Background:**

Taeniodonta is a clade of Late Cretaceous – Paleogene mammals remarkable for their relatively extreme cranial, dental, and postcranial adaptations and notable for being among the first mammals to achieve relatively large size following the Cretaceous-Paleogene mass extinction. Previous workers have hypothesized that taeniodonts can be divided into two clades: Conoryctidae, a group of small-bodied taeniodonts with supposedly “generalized” postcranial skeletons, and Stylinodontidae, a group of large-bodied, robust animals with massive forelimbs and claws adapted for scratch-digging. However, many taeniodont taxa are poorly known and few are represented by postcranial material, leaving many details about their anatomy, biology, and evolution ambiguous.

**Methodology/Principal Findings:**

In this paper, we describe three new specimens of the rare taxon *Wortmaniaotariidens* from the early Paleocene (Puercan) of New Mexico. Among these specimens is one that includes remarkably complete cranial and dental material, including associated upper and lower teeth, and another that consists of partial forelimbs. These specimens allow for an updated anatomical description of this unusual taxon, supply new data for phylogenetic analyses, and enable a more constrained discussion of taeniodont biology and functional morphology.

**Conclusions/Significance:**

The new specimen of *Wortmania* that includes associated upper and lower teeth indicates that previous interpretations of the upper dentition of this taxon were not accurate and the taxon *Robertschochiasullivani* is a junior synonym of *W* *. otariidens* . New specimens that include partial forelimbs indicate that *Wortmania* is very similar to later, large-bodied taeniodonts, with marked and distinctive adaptations for scratch-digging. Comparisons with other taeniodont taxa that include postcranial material suggest that all taeniodonts may have had scratch-digging adaptations. A phylogenetic analysis shows that *Schowalteria* and *Onychodectes* are basal taeniodonts, Stylinodontidae (including *Wortmania*) is monophyletic, and a monophyletic Conoryctidae (but not including *Onychodectes*) is only recovered when certain characters are ordered.

## Introduction

During the first ~10 million years after the Cretaceous–Paleogene mass extinction, the recovery and re-radiation of mammals was driven by the diversification of several “archaic” groups: clades that do not persist into the modern world and whose relationships with extant groups are debatable [1]. Among these “archaic” groups are the taeniodonts, a clade known only from the Cretaceous – Paleogene of North America (see [2,3] for summaries). Taeniodonts were among the first mammals to achieve relatively large size following the mass extinction of the dinosaurs, were the first group of mammals to evolve crown hypsodonty, and exhibited a range of cranial and postcranial features that suggest a diversity of feeding styles and behaviors compared to contemporary mammals, including the ability to scratch-dig and eat abrasive prey [2,4].

A better understanding of taeniodont anatomy, phylogeny, biology, and evolution may hold a key to better understanding the tempo and structure of the post-Cretaceous blossoming of mammals. Unfortunately, taeniodonts are rare components of Cretaceous and Paleogene faunas and many taxa are very poorly known, represented by few specimens that include only portions of the dentition. Among the rarest and most unusual taeniodonts is *Wortmaniaotariidens* , a relatively large taxon referred to the subgroup Stylinodontidae, a clade characterized by large size, massive jaws with enlarged canines, and adaptations for digging that include powerful forelimbs with large, deep, and laterally compressed claws. *Wortmania* was long regarded as the earliest and most primitive member of Stylinodontidae and the only member of the subclade to be present in the earliest Paleocene middle and late Puercan North American land-mammal “age” [5]. Recent discoveries have revealed greater stylinodontid diversity in the latest Cretaceous and earliest Paleocene [6,7], but *Wortmania* remains a vital taxon in understanding the origins of taeniodonts specifically and the initial phase of mammalian diversification after the dinosaur extinction more generally [3,8].

**Figure 1:**
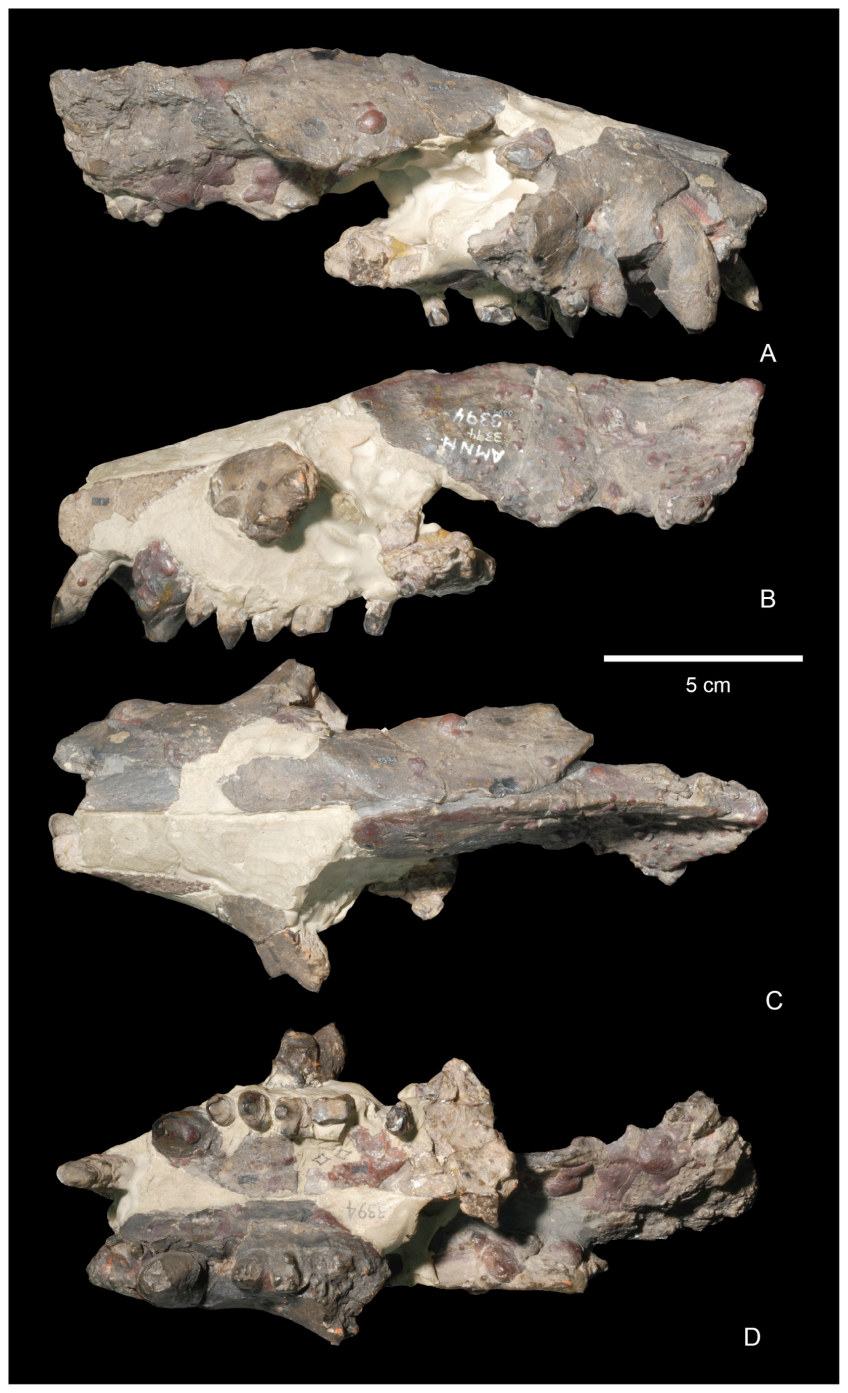
Skull of the lectotype of *Wortmaniaotariidens* (AMNH 3394). A, right lateral view; B, left lateral view; C, dorsal view; D, palatal view.

**Figure 2:**
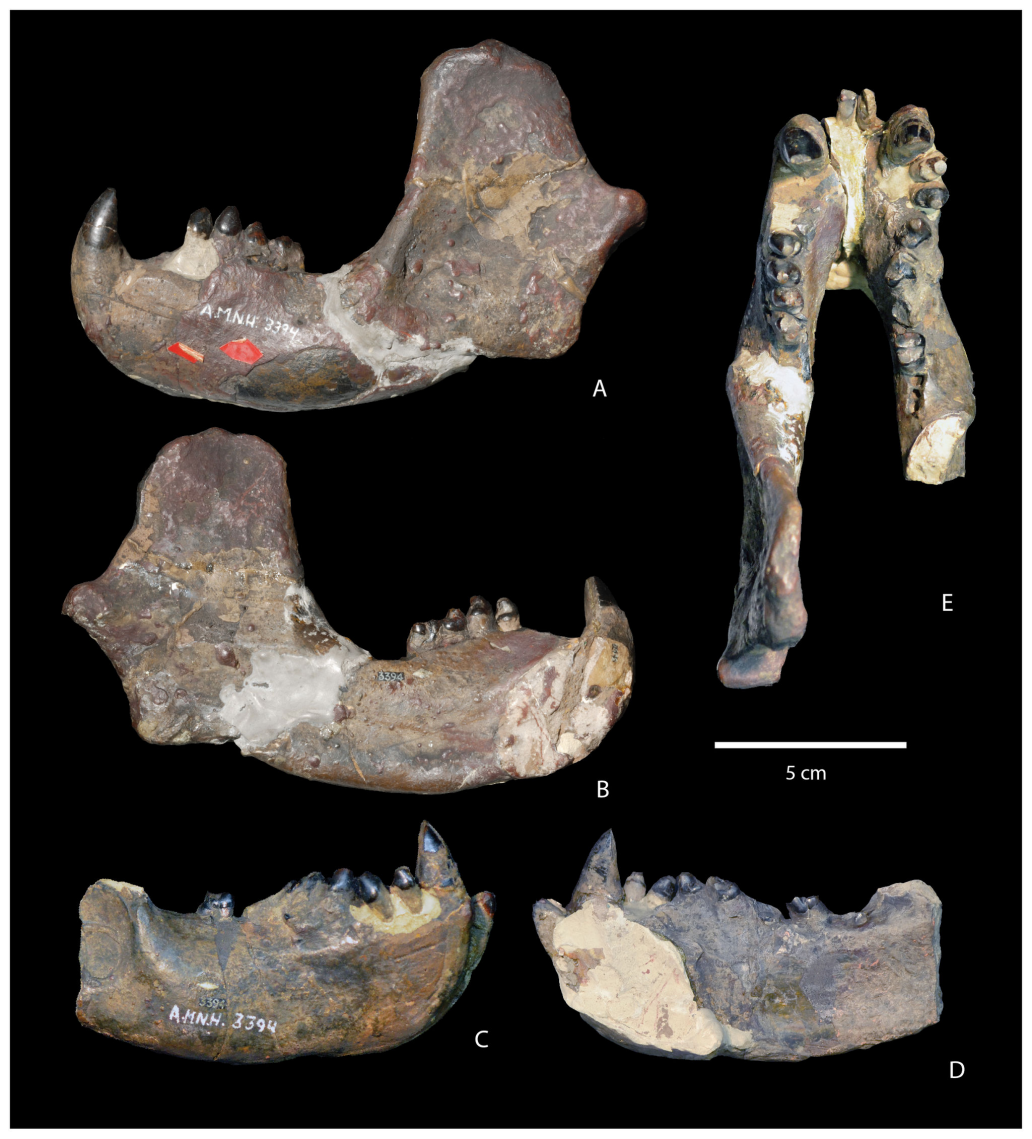
Mandible of the type of *Wortmaniaotariidens* (AMNH 3394). Left dentary in lateral (A) and medial (B) views. Right partial detnary in lateral (C) and medial (D) views. Left and right dentaries in occlusal view E.

**Figure 3:**
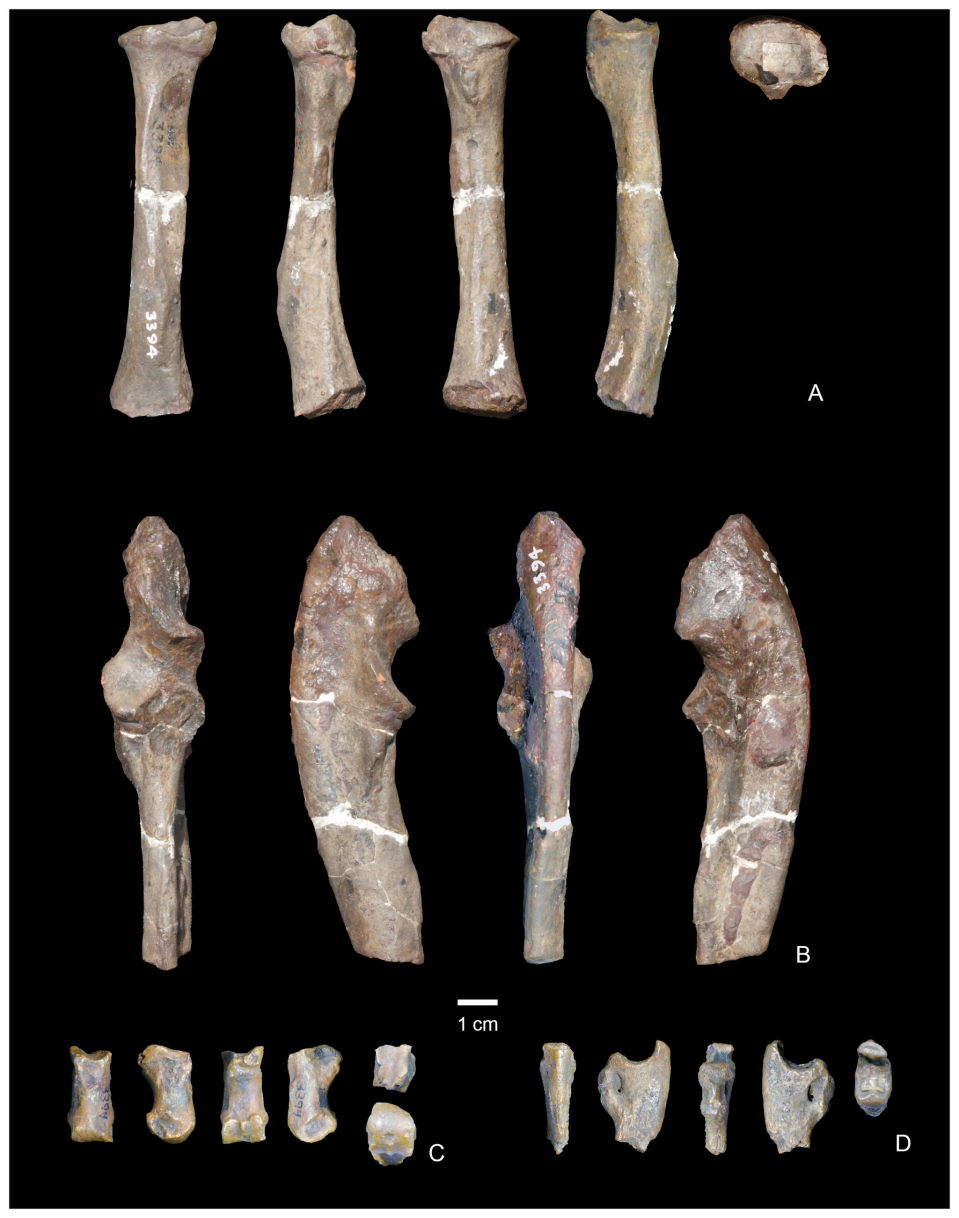
Forelimb of the type of *Wortmaniaotariidens* (AMNH 3394). Left partial radius in anterior, medial, ventral, lateral, and proximal views (A). Left partial ulna in anterior, medial, ventral, and lateral views (B). Right metacarpal II in dorsal, lateral, ventral, medial, proximal, and distal views (C). Ungual phalanx in dorsal, lateral?, ventral, medial?, and proximal views (D).

Known specimens of *Wortmania* come exclusively from the Nacimiento Formation of the San Juan Basin, New Mexico, which contains the best record of taeniodonts from the early Paleocene, when the clade peaked in taxonomic diversity. The type of *Wortmania*, American Museum of Natural History (AMNH) 3394, a specimen collected by David Baldwin for E. D. Cope in 1885 from the Nacimiento Formation, includes a partial skull (Figures 1-2) and skeleton, the only postcrania documented for this taxon [2]. This specimen revealed an anatomically peculiar and relatively large animal, one of the largest animals known from the early Paleocene (estimated body mass of up to ~30 kilograms [2]), with “robust proportions, and with a wide head an exceedingly short thick muzzle, armed with some formidable teeth in front” ( [9]: 311). Slightly over a decade after its initial discovery, Wortman [10] provided a more detailed description of the unusual *Wortmania* type (p. 68) “…the face is short, the sagittal crest is long and not very prominent, the lower jaw is short, deep and robust…” Wortman [10] also first noted that the mandibular condyle is positioned high above the tooth row. Wortman [10] provided the first significant description of the associated forelimb bones (Figure 3), describing the partial ulnae, nearly complete radius, lunar, second metacarpal (Cope [9] had identified this bone as a metatarsal) and an ungual. The radius was described as short and robust with humeral and ulnar articulations that permitted (p. 69) “free pronation and supination of the manus.” Wortman [10] also provided the first detailed description of the metacarpal II and ungual, recognizing the relatively short length of the metacarpal and large, laterally compressed ungual, with its deeply excavated proximal articular surface, and large, plantar tuberosity.

Since the discovery of the type, additional material of *Wortmania* has been discovered, but most of this is exceedingly incomplete [2]. Furthermore, considerable debate has focused on the dentition of this taxon over the past century. New specimens have been unable to shed much light on this issue, and as a result, several major questions about the craniodental anatomy of *Wortmania* remain unanswered. The type includes the skull (Figure 1) and mandibles (Figure 2) with parts of the upper dentition including an upper incisor, the canines, and damaged and fragmentary upper premolars and molars and most of the lower dentition. The incisors were originally found loose and each exhibited a long, tapering, and closed root [10]. Wortman [10] also described the incisors as having enamel limited to the anterior face of the crown. The canines are large, with a similar long, tapering, and closed root [10]. The crown of the lower canines is worn and enamel is restricted to the anterior face of the crown. It cannot be determined if enamel originally completely encircled the lower canines, but Wortman [10] suggested that newly erupted teeth may have started that way, with just a thin layer of enamel over the posterior face. Wortman [10] described the upper canines as possessing enamel over the entire crown, but with only a very thin layer of enamel over the posterior face.

In early descriptions of the type specimen [9-12], little could be described of the morphology of the upper cheek teeth because they are highly worn and because most of the teeth were not found in place, and so their loci could not be ascertained. Most problematic, long-standing disagreement concerned one of the most fundamental properties of the dentition: the number of incisors and premolars possessed by *Wortmania*. Wortman [10] believed that two pairs of incisors were present whereas Matthew [11] and Patterson [12] concluded that *Wortmania* had merely a single pair. Furthermore, Matthew [11] believed that *Wortmania* had three upper premolars, but Patterson [12] argued that it had four. Patterson’s [12] conclusions were presented with composite drawings of the upper dentition of *Wortmania* (Patterson [12]: fig. 1a) based on specimens at the AMNH and the USNM, probably incorporating specimen USNM 15429, a partial maxilla that includes two worn teeth. He apparently interpreted these two teeth as representing M1-M2.

Nearly 40 years later, Schoch [2] documented and illustrated all *Wortmania* specimens known at that time. Of these, other than the type, only two specimens include what may be upper molars: AMNH 16342, a fragment of a maxilla contains a single incomplete unworn molar (see Schoch [2]: Plate 17, fig. 5) representing a M1 or M2 and USNM 15429. Schoch interpreted the two worn teeth of USNM 15429 to possibly represent a P4 and M1 (see Schoch [2]: Plate 17, fig. 7). Schoch [2] also noted that since Matthew’s [11] description of the type, the originally loose upper cheek teeth and several teeth of the lower dentition were set in plaster and might not be in their original positions. Schoch [2] evidently based his restoration of some of the upper cheek teeth of *Wortmania* (Schoch [2]: fig. 11) on USNM 15429. He also concluded that *Wortmania* had at least three upper premolars and possibly four, and an undetermined number of upper molars.

Therefore, despite its importance as an early taeniodont and one of the first examples of a moderately large-bodied and ecologically specialized post-Cretaceous mammal, *Wortmania* remains a largely mysterious taxon. More than anything, this is due to the great rarity of fossil specimens. Here, we describe three new specimens of *Wortmaniaotariidens* from the Nacimiento Formation, San Juan Basin, New Mexico, which reveal important new anatomical information for this puzzling taxon. One of these specimens (NMMNH P-64001) includes a partial skull and mandibles and an associated and nearly complete upper and lower dentition, making it the most complete specimen of *Wortmania* to be collected in over 100 years. Additional new specimens include portions of left and right forelimbs of a single individual (NMMNH P-19460) that represent the only postcranial bones referred to *Wortmania* other than the type. A third specimen (NMMNH P-64929) includes fragments of a lower canine and an upper incisor. These specimens provide critical new information on the dentition and postcranial anatomy of *Wortmania*, which allow us to make detailed comparisons to other taeniodonts. These comparisons help resolve the alpha-level systematic of stylinodontids (particularly the systematic affinities of the poorly known and possibly congeneric *Robertschochia*), provide new data that can be incorporated into phylogenetic analyses, and enable more detailed discussions of the functional anatomy and biology of taeniodonts.

### Geologic Setting

*Wortmaniaotariidens* is known with certainty only from the Nacimiento Formation, a primarily fluvial deposit found in the Laramide San Juan Basin of northwestern New Mexico [13]. The Nacimiento Formation contains the type faunas of the Puercan and Torrejonian land-mammal “ages” [5]. All *Wortmania* specimens for which there is adequate locality information were recovered from near the base of the Nacimiento Formation from fossil zones that yield either middle or late Puercan faunas [5,13]. All Wortmania specimens were collected using surface-collecting techniques.

Lower Nacimiento Formation deposits are primarily fluvial deposits interspersed with moderately to well-developed paleosols and thin carbonaceous shale units [13]. The presence of crocodilians and palms in the lower Nacimiento Formation indicates that Puercan faunas of the Nacimiento Formation enjoyed a frost-free climate. The composition of the Nacimiento flora is consistent with warm to hot and humid conditions [14].

### Materials and Methods

New Mexico Museum of Natural History and Science (NMMNH) specimens are accessioned into the Geoscience Collections of the New Mexico Museum of Natural History and Science, an institution accredited by the American Association of Museums. Access to precise locality information is restricted to qualified researchers and land management personnel.

Collection of NMMNH P-64001 was conducted under BLM Paleontological Resources Use Permit NM11-003S NLCS BDNZ. Collection of NMMNH P-19460 was conducted under BLM Federal Antiquities Permit 1-P-NM-91-A and Paleontological Resources Use Permit NMMNH-8270-RS-00-1. Collection of NMMNH P-64929 was collected under BLM Paleontological Resources Use Permit NMMNHS-8270-RS-0011-1.

Cranial measurements are provided in Table 1, mandibular measurements in Table 2, and dental measurements in Table 3. Measurements of postcrania are provided in Table 4.

**Table 1:** Selected measurements of the skull of *Wortmaniaotariidens* (NMMNH P-64001).

**Element**	**Measurement**
Maxilla, height of zygomatic arch at root	12.7
Maxilla, mediolateral width from P3 to midline	26.3
Maxilla, mediolaterial width of nasal pharynx from lateral wall to midline	10.5
Squamosal, height lateral to the glenoid	10.7
Squamosal, anteroposterior diameter of glenoid	12.8

Measurements are in mm.

**Table 2:** Selected measurements of the mandible of *Wortmaniaotariidens* (NMMNH P-64001).

**Dentary**	**Measurement**
Length	137.6
Depth below p1	36.0
Depth below m2	30.2
Mandibular symphysis maximum diameter	42.5*
Mandibular symphysis minimum diameter	24.3
Maximum height of coronoid process	78.8*

Measurements are in mm. An asterisk indicates the measure is approximate.

**Table 3:** Selected measurements of the dentition of *Wortmaniaotariidens* .

**Tooth**	**Measurement**
I (NMMNH P-64001), length	4.8
I (NMMNH P-64001), width	6.6
C1 (NMMNH P-64001), length	10.3
C1 (NMMNH P-64001), width	12.1*
P1 (NMMNH P-64001), length	5.9*
P1 (NMMNH P-64001), width	6.5
P2 (NMMNH P-64001), length	6.7
P2 (NMMNH P-64001), width	8.8
P3 (NMMNH P-64001), length	7.0*
P3 (NMMNH P-64001), width	11.3
P3 (NMMNH P-9000), length	6.3
P3 (NMMNH P-9000), width	9.1
P4 (NMMNH P-64001), length	6.8
P4 (NMMNH P-64001), width	10.7
P4 (NMMNH P-9000), length	6.1
P4 (NMMNH P-9000), width	10.5
M1 (NMMNH P-64001), length	7.5
M1 (NMMNH P-64001), anterior width	13.4
M1 (NMMNH P-64001), posterior width	12.9
M1 (NMMNH P-9000), length	6.5
M1 (NMMNH P-9000), anterior width	11.8
M1 (NMMNH P-9000), posterior width	11.7
M2 (NMMNH P-64001), length	6.6
M2 (NMMNH P-64001), anterior width	11.6
M2 (NMMNH P-64001), posterior width	10.8
M2 (NMMNH P-9000), length	6.0
M2 (NMMNH P-9000), anterior width	12.4
M2 (NMMNH P-9000), posterior width	11.1
i (NMMNH P-64001), length	5.2
i (NMMNH P-64001), width	9.8
c1 (NMMNH P-64001), length	11.5
c1 (NMMNH P-64001), width	15.2*
c1 (NMMNH P-64929), length	11.1
p1 (NMMNH P-64001), length	6.6*
p1 (NMMNH P-64001), width	10.2*
p2 (NMMNH P-64001), length	6.9
p2 (NMMNH P-64001), width	9.5
p3 (NMMNH P-64001), length	7.3
p3 (NMMNH P-64001), width	9.1
p4 (NMMNH P-64001), length	7.8
p4 (NMMNH P-64001), width	7.4
m1 (NMMNH P-64001), length	8.5
m1 (NMMNH P-64001), anterior width	7.7
m1 (NMMNH P-64001), posterior width	7.0
m2 (NMMNH P-64001), length	8.3
m2 (NMMNH P-64001), anterior width	7.3
m2 (NMMNH P-64001), posterior width	6.1

Measurements are in mm. An asterisk indicates the measure is approximate.

**Table 4:** Selected measurements of the postcrania of *Wortmaniaotariidens* .

**Element**	**Measurement**
Ulna (NMMNH P-19460), length of semilunar notch	24.5*
Ulna (NMMNH P-19460), depth of semilunar notch	6.2*
Ulna (NMMNH P-19460), maximum mediolateral breadth	21.5
Radius (NMMNH P-19460), length	93.0
Radius (NMMNH P-19460), anteroposterior midshaft diameter	11.0
Radius (NMMNH P-19460), midshaft mediolateral diameter	11.5
Metacarpal II (NMMNH P-19460), length	25.0
Metacarpal II (NMMNH P-19460), proximal breadth	9.8
Metacarpal II (NMMNH P-19460), midshaft mediolateral diameter	8.7
Metacarpal II (NMMNH P-19460), distal breadth	12.4
Metacarpal III (NMMNH P-19640), length	29.3
Metacarpal III (NMMNH P-19640), proximal breadth	13.9
Metacarpal III (NMMNH P-19640), midshaft mediolateral diameter	9.8
Metacarpal III (NMMNH P-19640), distal breadth	13.5
Metacarpal IV (NMMNH P-19640), length	23.9
Metacarpal IV (NMMNH P-19640), proximal breadth	10.9
Metacarpal IV (NMMNH P-19640), midshaft mediolateral diameter	6.8
Metacarpal IV (NMMNH P-19640), distal breadth	9.9
Ungual (NMMNH P-19640), proximal breadth of most complete	8.6

Measurements are in mm. An asterisk indicates the measure is approximate.

Directional references related to the forelimb are based on the forelimb in a pronated position as in the dog. Anatomical references follow “Miller’s Anatomy of the Dog” [15].

In some figures, some specimens or portions of specimens have been dusted with magnesium oxide or ammonium chloride to bring out surface details.

## Results

### Systematic Paleontology

Mammalia Linnaeus 1758 [16].
Eutheria Gill 1872 [17].
Taeniodonta Cope, 1876 [18].
Stylinodontidae Marsh, 1875 [19].
*Wortmania* Hay, 1899 [20].

#### Emended diagnosis

*Wortmania* differs from all other known taeniodonts by a combination of character states. In this section, it is compared first with stylinodontid and then non-stylinodontid taeniodonts, and each comparison is organized sequentially to cover cranial, dental, and postcranial characters. *Wortmania* differs from *Psittacotherium*, *Ectoganus*, and *Stylinodon* because of its smaller size and less robust skeleton, with enamel extending around the lingual margin of the lower incisors, canines relatively smaller, first premolars relatively smaller, upper molar ectocingulum present, upper molars more transverse, crown hypsodonty less pronounced, mandibular symphysis unfused, manus and forelimb are less robust with relatively less developed muscle attachment sites. Differs from *Ectoganus* and *Stylinodon* in lacking rootless teeth and anterior and posterior walls of teeth lacking thick covering of cementum. Differs from *Psittacotherium*, *Ectoganus* and *Stylinodon* in that the mastoid process is relatively smaller. Differs from *Ectoganus* and *Stylinodon* in the mastoid process is less mediolaterally elongate. Differs from *Psittacotherium* and *Stylinodon* in lacking a distally-extending, finger-like projection of the styloid process. Differs from *Onychodectes* and *Conoryctes* in that the mastoid process is positioned posterolateral to the glenoid process. Differs from *Schowalteria*, *Onychodectes*, *Conoryctella*, and *Conoryctes* in having a single pair of upper and lower incisors. Differs from *Schowalteria*, *Alveugena*, *Onychodectes*, *Conoryctella*, *Conoryctes*, in having relatively larger canines with rugose enamel texture. Differs from *Alveugena*, *Onychodectes*, *Conoryctella*, and *Conoryctes* in that its lower canines are more elongate and develop a posterior platform. Differs from *Schowalteria* and *Alveugena* in having upper molars progressively reducing in size from M1 through M3 and M2 parastyle positioned mesial to paracone. Differs from Schowalteria, *Onychodectes*, *Conoryctella*, and *Conoryctes* in lower incisors resembling canines in being labiolingually elongate rather than circular in cross section at base. Differs from *Schowalteria*, *Alveugena*, *Onychodectes*, *Conoryctella*, *Conoryctes*, and *Huerfanodon* in having larger and relatively deeper mandibles. Differs from *Schowalteria*, *Onychodectes*, *Conoryctella*, *Conoryctes*, and *Huerfanodon* in having a p4 paraconid that is relatively larger and better-developed. Differs from *Schowalteria*, *Alveugena*, *Onychodectes*, *Conoryctella*, *Conoryctes*, and *Huerfanodon* in having more transverse P2-P3 with large protocones on P2. Differs from *Conoryctes* in that P1 is present. Differs from *Schowalteria*, *Alveugena*, and *Onychodectes* in having a relatively larger P1 and differs from *Schowalteria*, *Alveugena*, *Onychodectes*, and *Conoryctes* in having a relatively larger p1 that is oriented obliquely. Differs from *Onychodectes*, *Conoryctella*, *Conoryctes*, and *Huerfanodon* in having upper molar para- and metacones conjoined at base and a mesiodistally more compressed talon. Differs from *Conoryctes* and *Huerfanodon* in that the upper molars lack mesostyle. Differs from *Onychodectes* in having more robust fore- and hindlimbs with relatively larger and deeper manual unguals.

*Hemiganus*: Wortman, p. 67 [10].
1899 *Wortmania* Hay, p. 593 [20].
1937 *Wortmania* Matthew, p. 270 [11].
1993 *Schochia* Lucas and Williamson, p. 175 [6].
1998 *Schochia* Lucas et al., p. 263 [3].
2011 *Robertshochia* Lucas, p. 1216 [21].

#### Type species

*Hemiganusotariidens* Cope [22].

#### Diagnosis

As for the only known species [21].

**Figure 4:**
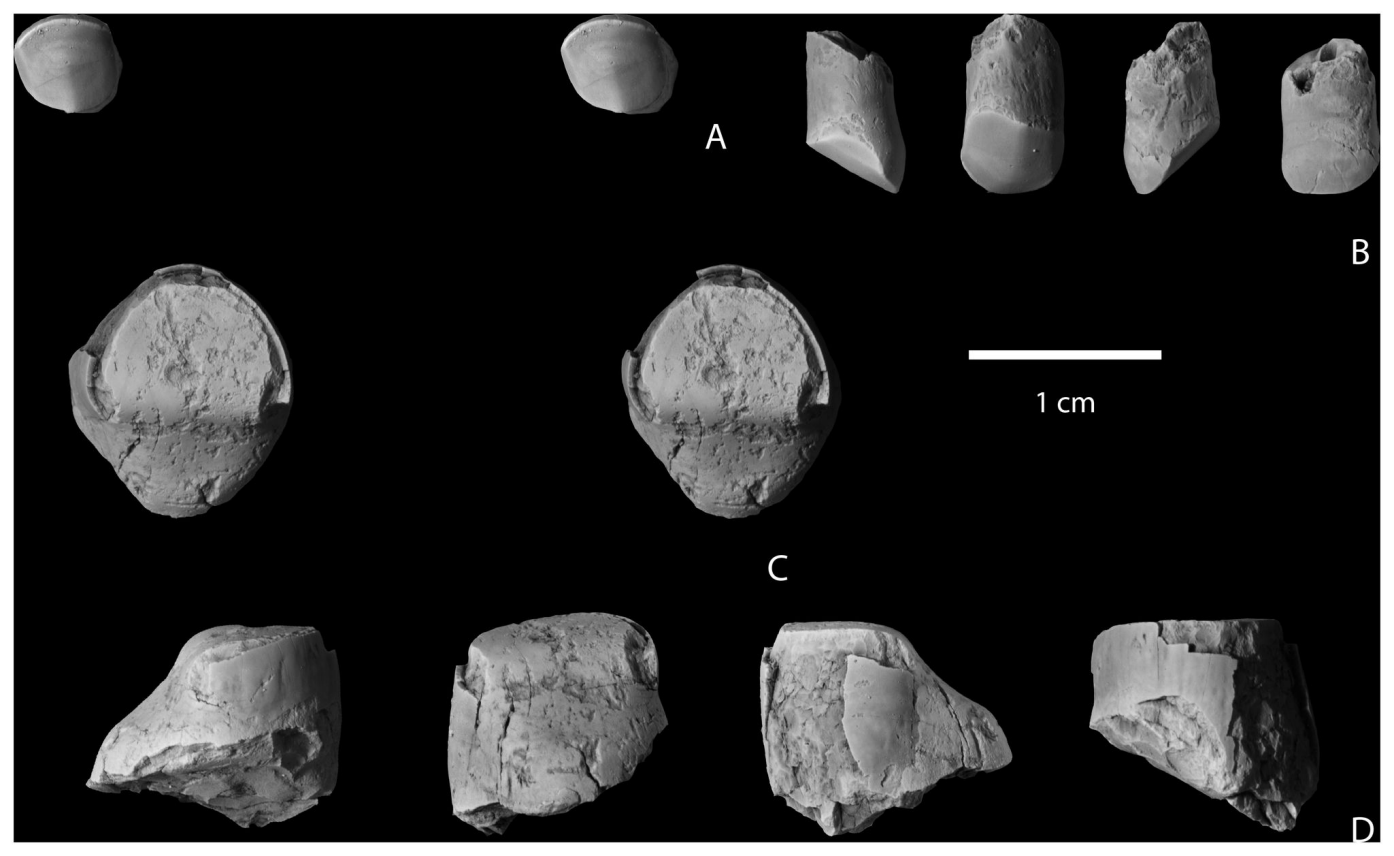
Upper incisor and partial lower canine of *Wortmaniaotariidens* (NMMNH P-64929). Upper incisor in occlusal (A, stereopair) and distal?, lingual, medial?, and labial views (B). Lower canine in occlusal (C, stereopair) and distal?, lingual, medial?, and labial views (D).

*Wortmaniaotariidens* (Cope) [22].
1885 *Hemiganusotariidens* Cope, p. 432 [22].
1888 *Hemiganusotariidens* Cope, p. 311 [9].
1897 *Hemiganusotariidens* Wortman, p. 67, Figures 2-3, 11 [10].
1937 *Wortmaniaotariidens* Matthew, p. 271, Figures 68-69, Plate lxii, Figures 1-4; Plate lxiii, Figures 1-4 [11].
1993 *Wortmaniaotariidens* Lucas and Williamson, Figure 2, Table 1 [6].
1993 *Wortmaniaotariidens* Williamson and Lucas, p. 122 [23].
1996 *Wortmaniaotariidens* Williamson, p. 40 [13].
1998 *Wortmaniaotariidens* Lucas et al., p. 264 [3].
1993 *Schochiasullivani* Lucas and Williamson, p. 175, Figures 1-2, Table 1 [23].
1998 *Schochiasullivani* Lucas et al., p. 263 [3].
1993 *Schochiasullivani* Williamson and Lucas, p. 122 [23].
1996 *Schochiasullivani* Williamson, p. 40 [13].
2011 *Robertschochiasullivani* , Lucas, p. 1216 [21].

#### Lectotype

AMNH 3394, partial skull (Figure 1), mandibles (Figure 2), and postcranial skeleton including right C, P3-4, left I3?, C, and several upper teeth, some fragmentary, set in plaster. Lower dentition includes a left i, c, and four left cheek teeth. Schoch [2] identified these as representing only three teeth; p3-4, m1, and the alveoli for m2. We suggest that these teeth represent p2-m1, but the first two of these are set in plaster and may not be properly placed (see below for discussion); right i, c, p1-4, roots for m1, m2, and alveoli for m3. Postcrania includes a left half of the atlas, the central and right portion of the axis, three partial cervical vertebrae, partial left ulna, left radius, left metacarpal II, left lunar, manual ungual phalanx, partial right ulna (Figure 3), proximal half of left femur, and left tibia.

#### Previously referred specimens

AMNH 755, left c and bone fragments; AMNH 16432, left dentary fragment with roots of c, p1-2, roots of p3-4, and an associated maxilla with alveoli for M1, M2, and alveoli for M3; University of California Museum of Paleontology (UCMP) 36528, left dentary with roots of I, roots of c, p1-2, roots of p3-4; UCMP 89280, isolated p2 or p3; University of Kansas Museum of Natural History 12998, five premolars, right and left p1?, p2?, tooth fragment, and right m2; United States National Museum, National Museum of Natural History (USNM) 15428, right dentary with c; USNM 15429, left P3-4; USNM 17654, right P4?; USNM 17655, left P3? 

#### New referred specimens

NMMNH P-64929, NMMNH locality L-6387, “Black Toe locale”, West Flank Kimbeto Wash [13], fragments of a left lower canine. The specimen was associated with a highly worn, single-rooted tooth tentatively identified as an upper incisor (Figure 4).

**Figure 5:**
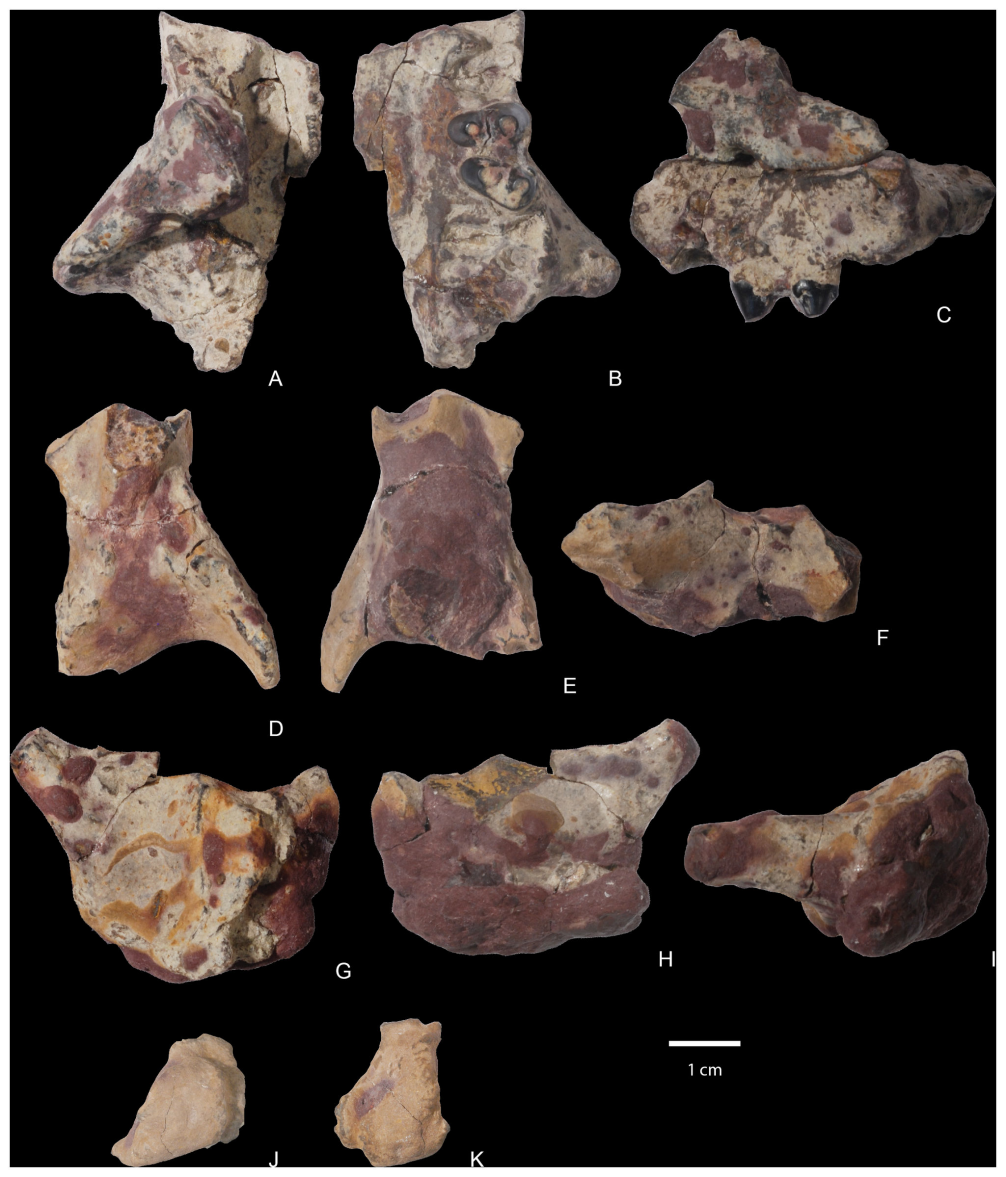
Partial cranium of *Wortmaniaotariidens* , NMMNH P-64001. Left maxilla in dorsal (A), ventral (B), and left lateral views. Right partial maxillae in dorsal (D) and ventral (E), and right lateral (F) views. Partial left squamosal in dorsal (G), ventral (H), and left lateral (I) views. Partial right basioccipital in posterior (J) and ventral (K) views.

**Figure 6:**
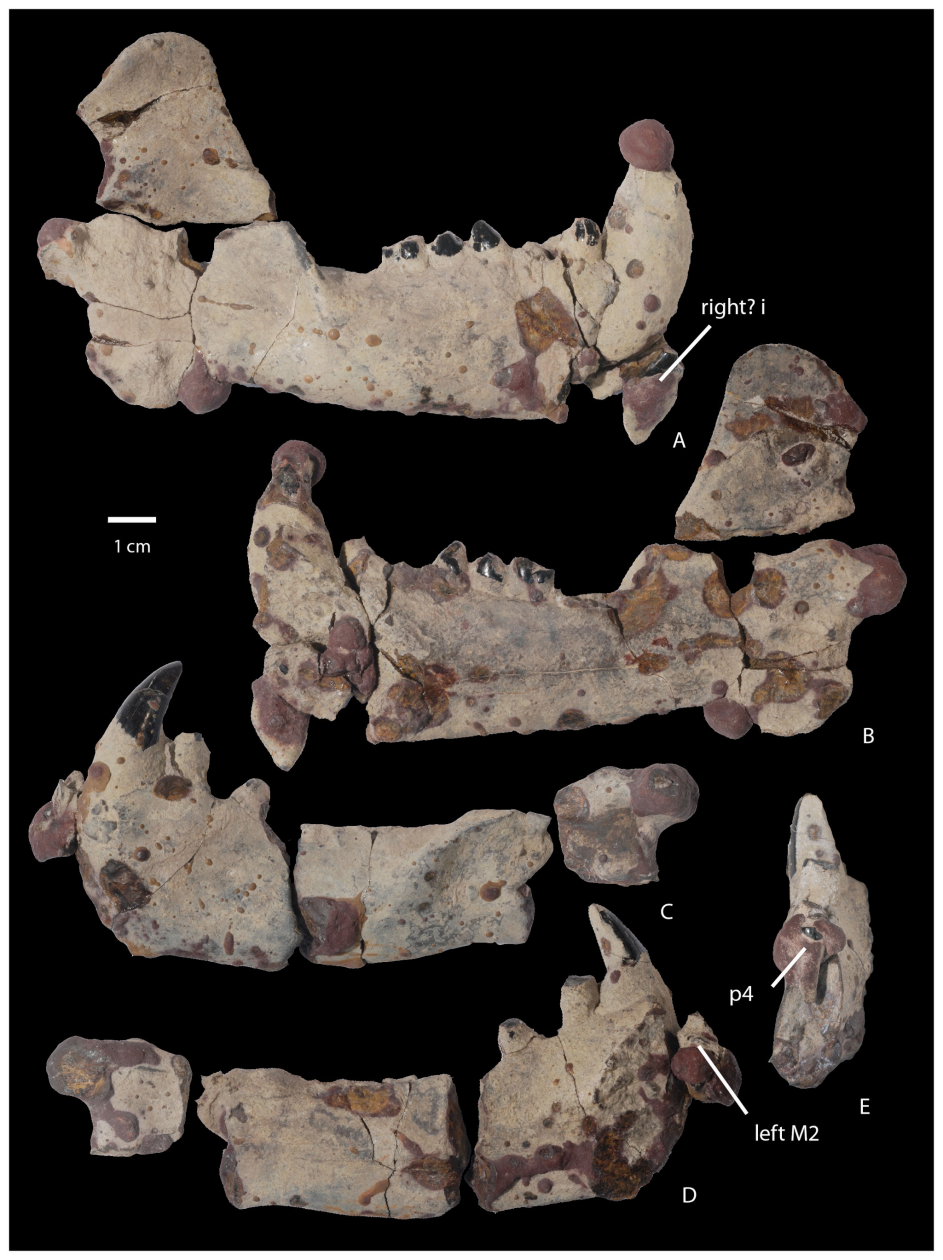
Mandibles of *Wortmaniaotariidens* , NMMNH P-64001. Right mandible with right? i?, c, p1, p3-m1 in lateral (A) and medial (B) views. Left partial mandible with c, partial p1, partial p3 in lateral (C) and medial (D) views (left M2 is cemented to left partial mandible with hematitic concretion; p4 and obscuring hematitic concretion is removed). Left anterior fragment of mandible in distal view with p4 and obscuring hematitic concretion in place (E).

**Figure 7:**
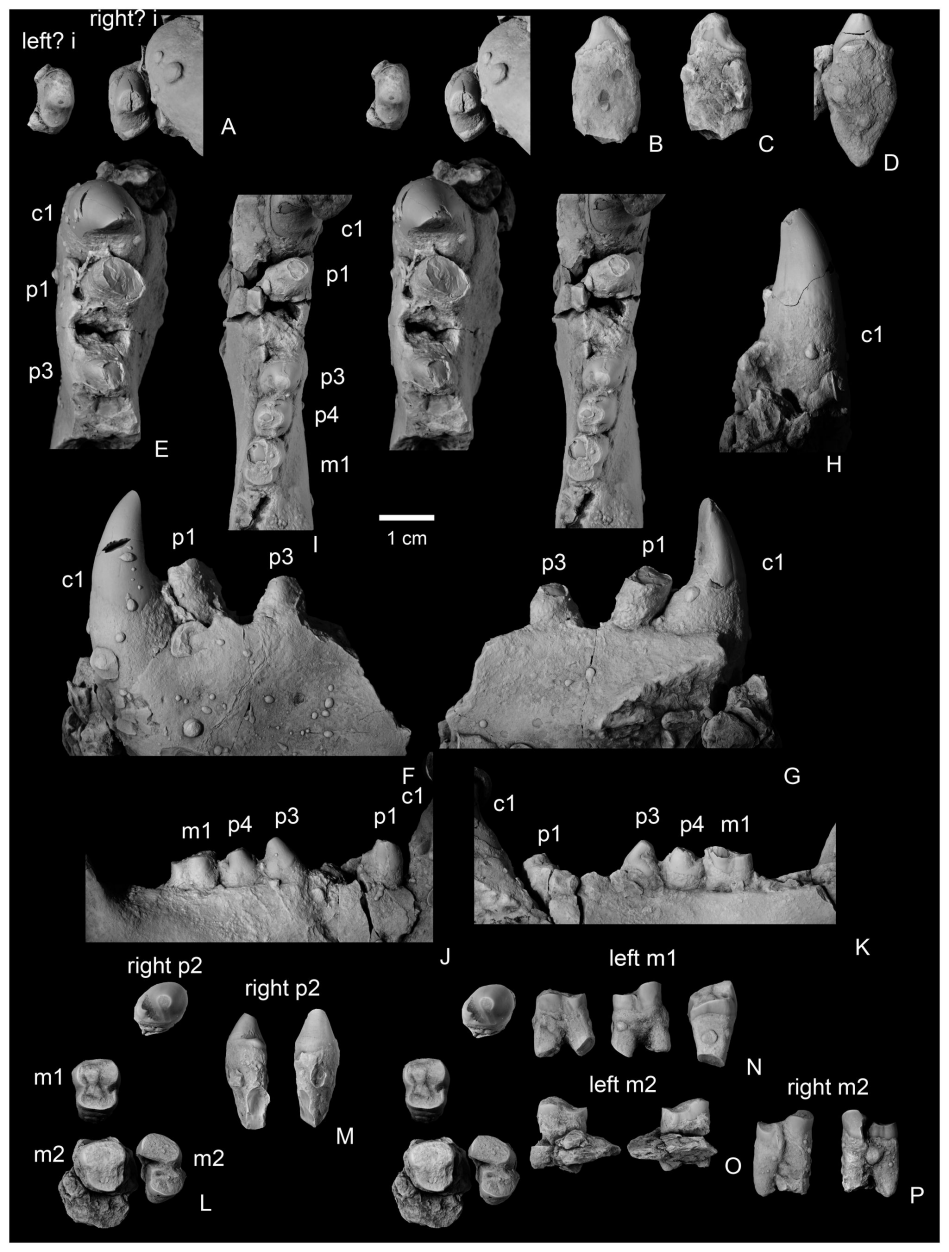
Lower dentition of *Wortmaniaotariidens* , NMMNH P-64001. Left? (left) and right? (right) i? in occlusal (A; stereopair). Left? i? in mesial (B) and distal (C) views. Right? i in distal (D) view. Partial left dentary with c, partial p1, alveolus for p2, and partial p3 (p4 and partially obscuring hematitic concretion is removed) in occlusal (E; stereo pair), buccal (F), medial (G), and anterior (H) views. Partial right dentary with c, p1, alveolus for p2, p3-m1, alveolus for m2 in occlusal (I; stereo pair), buccal (J), and medial (K) views. Right p2, left m1, left m2, and right m2 in occlusal (L; stereo pair). Right p2 in lingual and buccal views (M). Left m1 in buccal, lingual, and distal views (N). Left m2 in buccal and lingual views (O). Right m2 in buccal and lingual views (P). Left m1 and m2 in lingual (M) and buccal (N) views. Right p2 and right m2 in lingual (O) and buccal (P) views. Left m1 in posterior view (Q).

**Figure 8:**
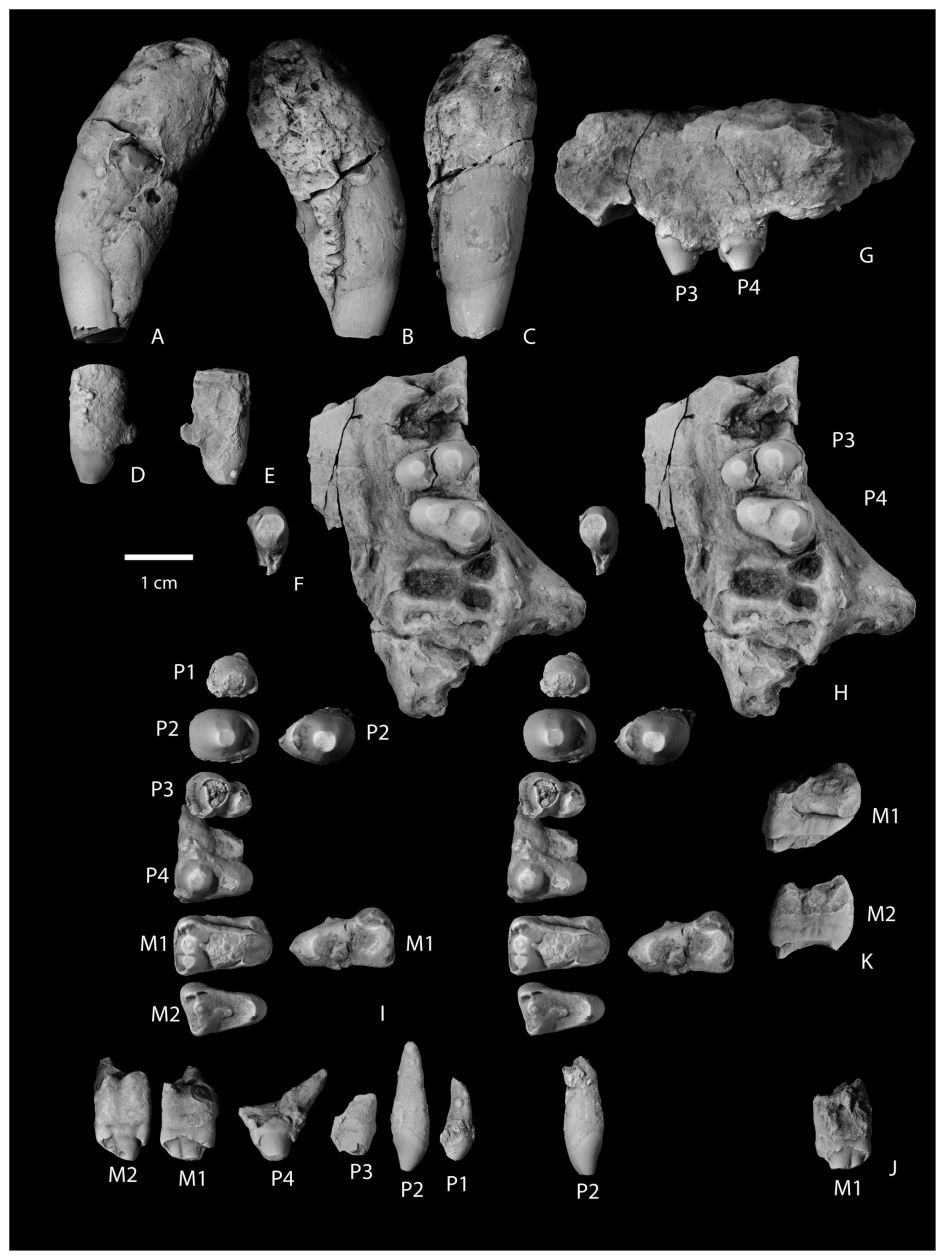
Upper dentition of *Wortmaniaotariidens* , NMMNH P-64001. Left partial upper canine in lateral (A), medial (B), and mesial (C) views. Left I in lateral (D), medial (E), and occlusal (F) views. Left maxilla with P3-P4 in buccal (G) and occlusal (H, stereo pair) views. Upper cheek teeth, right P1-M2 and left P2, M1 in occlusal (I; stereo pair) and buccal (J) views. Right M1 (upper) and M2 (lower) in distal view (K).

NMMNH P-64001, NMMNH locality L-8350, De-na-zin Wash locale [13]. This specimen (Figures 5-8) includes nearly complete left and right maxillae; a left squamosal; a right partial basioccipital with an occipital condyle; both dentaries; and several unidentified bone fragments that may be part of the same individual. Many of the bones are covered by a thin greenish encrustation as well as scattered irregular globules of reddish-purple hematite, a condition that is common for fossils from this locale. Identification of many of the smaller bone fragments from the site is hampered by their close association and admixing with many concreted bones of an alligatorid. The specimen is associated with most of the dentition. Numerous teeth are in their sockets and several teeth were loose and found closely associated with the rest of the specimen. Several teeth were found outside of their sockets but cemented to the dentaries by globules of hematite, and two remain in this position after the specimen has been prepared. The associated dentition includes teeth of the right dentary: a right? lower incisor (found loose and cemented to the right dentary by hematite), a lower damaged canine partly obscured by a globule of hematite, a first premolar, and p3-m1 in their sockets. A right p2 and an m2 were found loose. The m2 is fragmented and held together by a concretion. The left dentary is associated with a loose lower incisor, a canine, and a partial p1, and p3-p4 are preserved in their sockets. These premolars are damaged and partly obscured by globules of hematite. Left p2, m1, and m2 were found loose. The upper dentition includes loose right P2-M2. The P3 is slightly crushed and distorted and the P4-M1 are both partly obscured by encrusting hematite. The M1 is missing a portion of the lingual margin of the protocone. The left upper dentition includes a loose incisor, a canine missing its tip; a small, single-rooted tooth that is not well preserved but may represent a P1; and P3-P4, which are socketed in the left maxilla. Furthermore, M1 is loose and partly blanketed by hematite and the left M2 is cemented to the partial left dentary by concretion and largely obscured by hematite.

**Figure 9:**
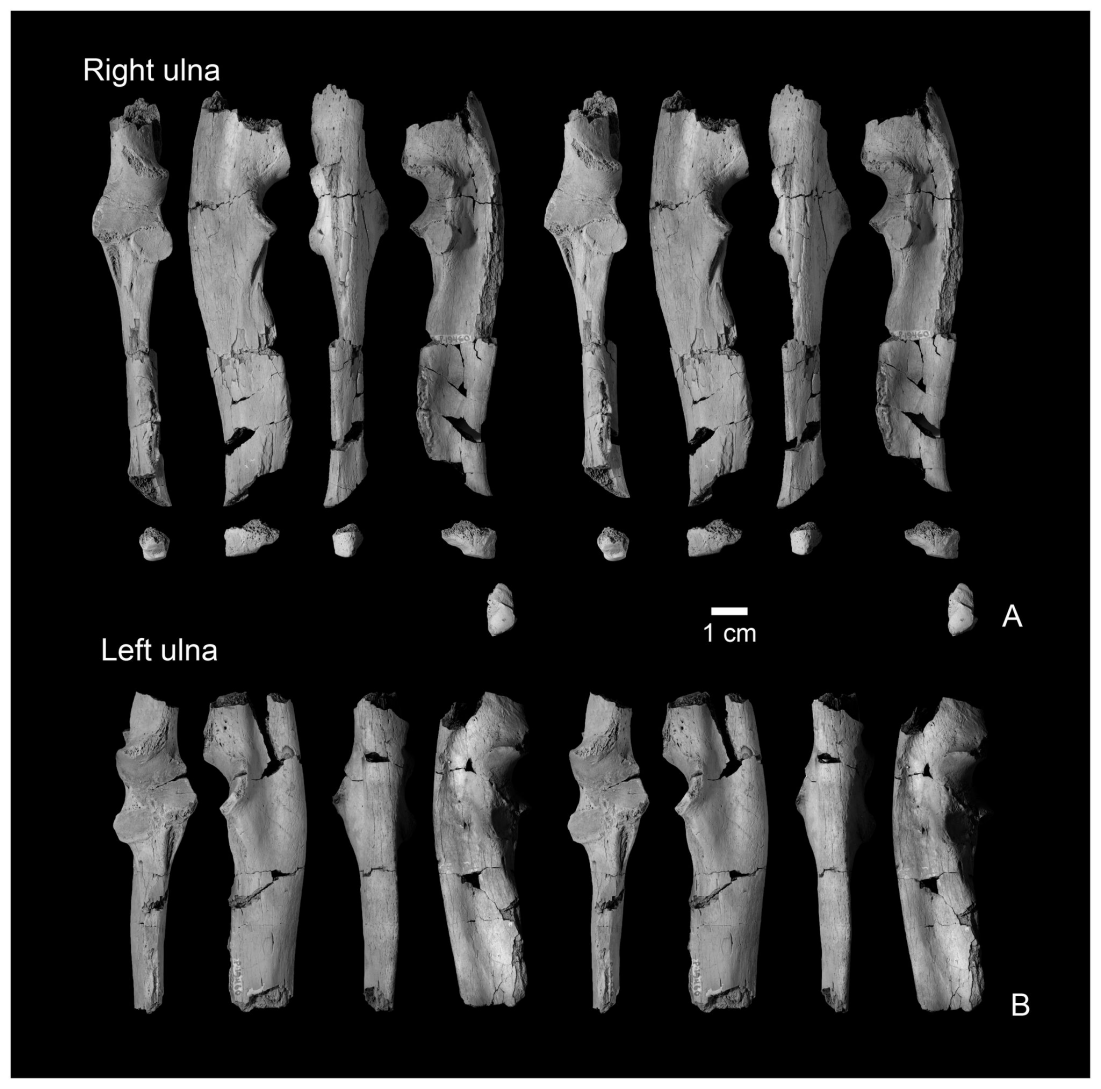
Ulnae of *Wortmaniaotariidens* , NMMNH P-19460. Right ulna in dorsal, lateral, ventral, medial, and distal views (A, stereo pairs). Left ulna in dorsal, lateral, ventral, and medial views (B, stereo pairs).

**Figure 10:**
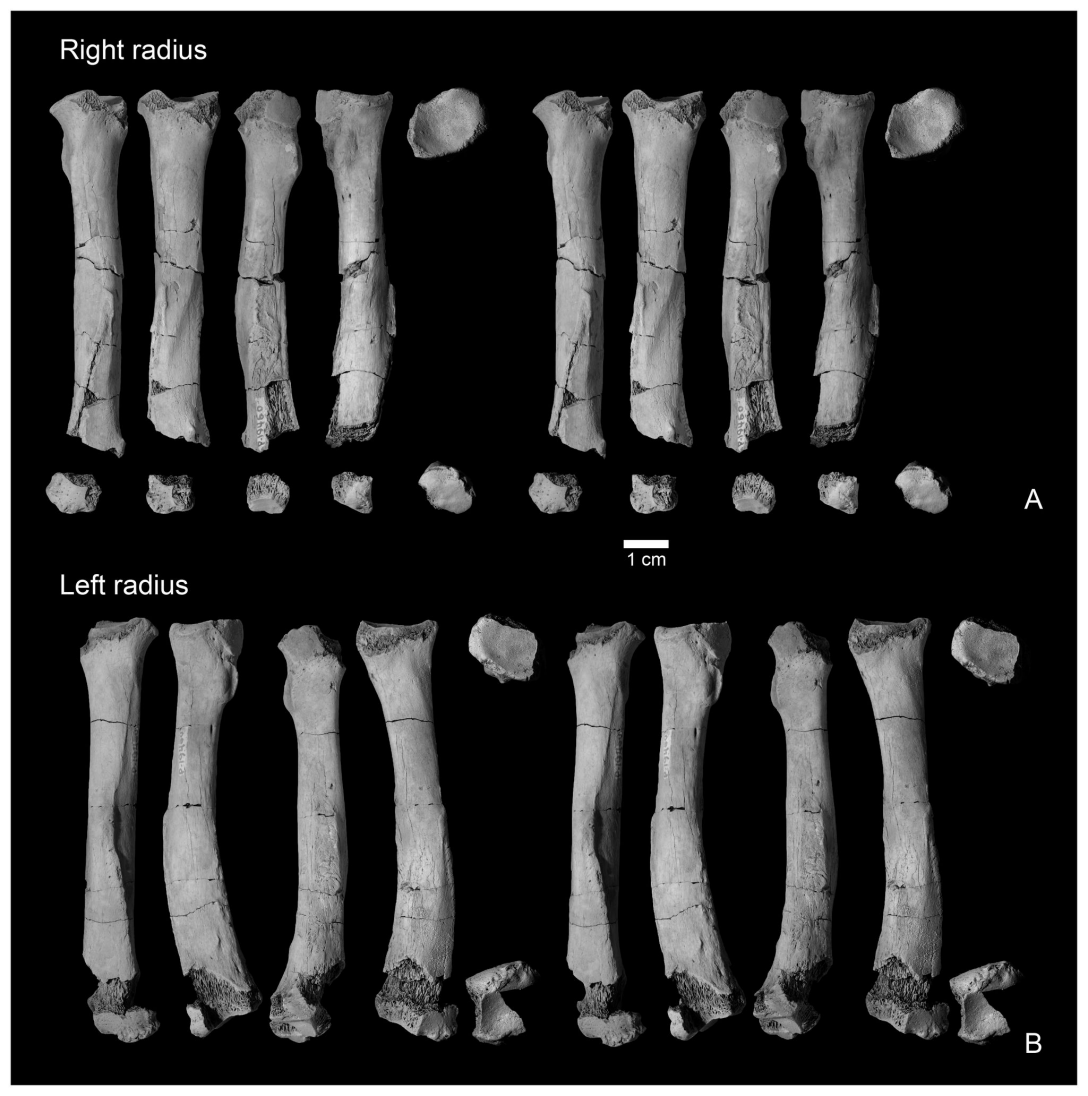
Radii of *Wortmaniaotariidens* , NMMNH P-19460. Right radius in dorsal, lateral, ventral, medial, proximal, and distal views (A, stereo pairs). Left radius in dorsal, lateral, ventral, medial, proximal, and distal views (B, stereo pairs).

**Figure 11:**
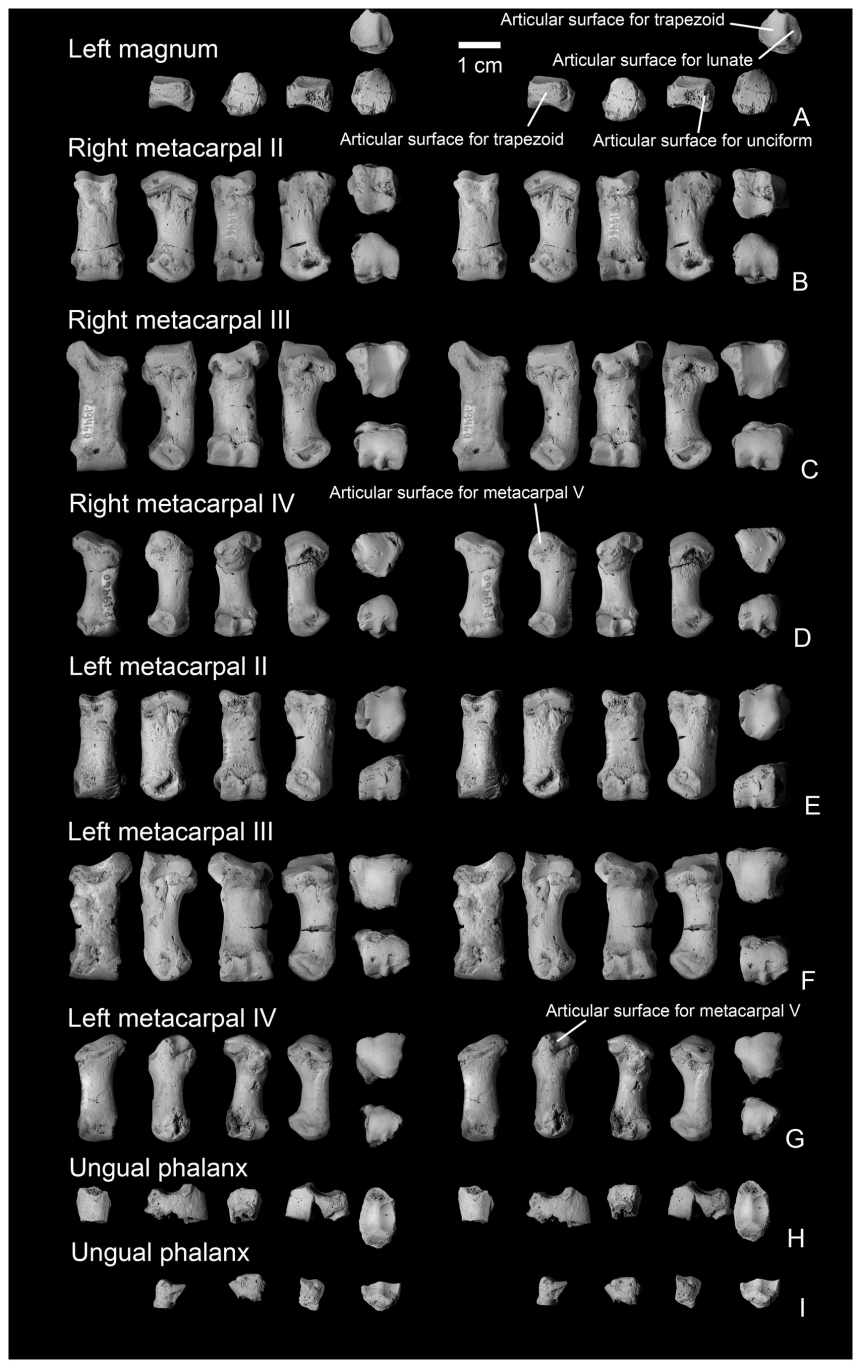
Mani of *Wortmaniaotariidens* , NMMNH P-19460. Partial left magnum in lateral, ventral, medial, proximal, and distal views (A, stereo pairs). Right metacarpal II in dorsal, lateral, ventral, medial, proximal, and distal views (B, stereo pairs). Right metacarpal III in dorsal, lateral, ventral, medial, proximal, and distal views (C, stereo pairs). Right metacarpal IV in dorsal, lateral, ventral, medial, proximal, and distal views (D, stereo pairs). Left metacarpal II in dorsal, lateral, ventral, medial, proximal, and distal views (E, stereo pairs). Left metacarpal III in dorsal, lateral, ventral, medial, proximal, and distal views (F, stereo pairs). Left metacarpal IV in dorsal, lateral, ventral, medial, proximal, and distal views (G, stereo pairs). Ungual phalanges in dorsal, lateral?, ventral, medial?, and proximal views (H-I, stereo pairs).

**Figure 12:**
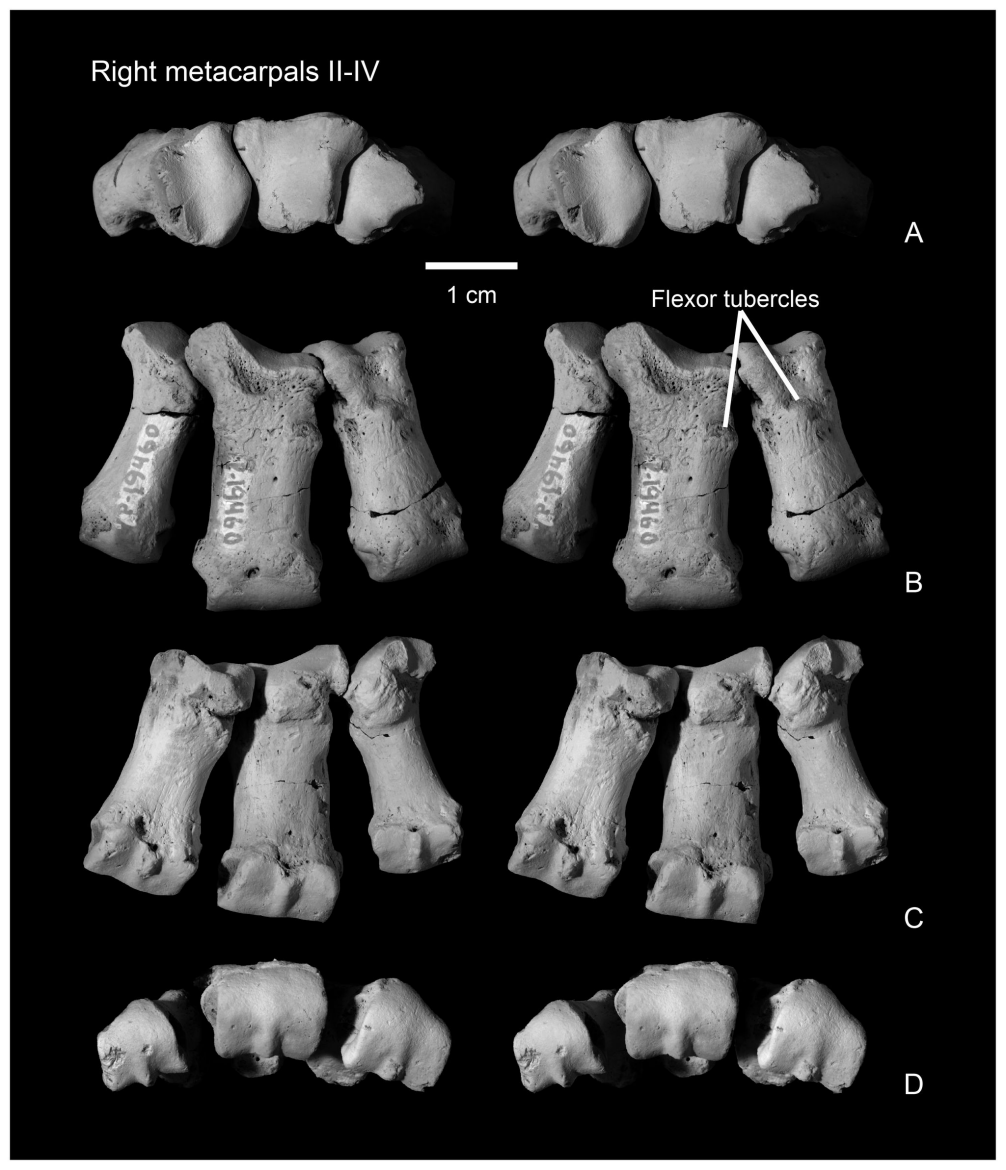
Articulated right metacarpals II-IV in proximal (A, stereo pair), dorsal (B, stereo pair), ventral (C, stereo pair), and distal (D, stereo pair) views.

NMMNH P-19460, NMMNH locality L-5203, West Flank Kimbeto Wash locale [13], includes left and right partial ulnae (Figure 9), left and right partial radiae (Figure 10), a partial left magnum, left and right metacarpals II, III, and IV, and two partial unguals (Figures 11-12).

#### Designation of lectotype

Herein we formally designate AMNH 3394 as the lectotype of *Wortmaniaotariidens* in accordance of Article 74 of the International Code on Zoological Nomenclature Code, in particular Article 74.6. Cope [22] did not designate a holotype specimen in his original description of *Hemiganusotariidens* . A partial skull and skeleton, identified as the specimen described by Cope [22] as *Hemiganusotariidens* , was first illustrated by Wortman ( [10]: Figures 1-2, 11). Wortman’s [10] inference that this specimen represents the type specimen is deemed to constitute lectotype fixation (International Commission on Zoological Nomenclature Article 74.6.1). This specimens was first identified by a specimen number (AMNH 3394) in Matthew [11].

### Description

#### Skull

Previously, the only known skull material of *Wortmania* was the highly fragmentary and eroded type specimen (AMNH 3394; Figures 1-2), which Matthew [11] and Schoch [2] could only describe in brief detail because of its poor state of preservation. Several portions of the skull are preserved in NMMNH P-64001 including regions of both maxillae, most of the left squamosal, and a portion of the basioccipital (Figure 5). This material helps clarify features of the type and allows for new anatomical observations, both of which enable a greater understanding of the cranial morphology of *Wortmania*. In general, this material corroborates Schoch’s [2] description of the skull of *Wortmania* as possessing a short and deep face anteriorly.

In NMMNH P-64001, the left maxilla is preserved as two fragments that were separated before burial (Figure 5A, C). The right maxilla (Figure 5A, D) is the less complete of the two specimens, as the alveoli and much of the palate are obscured by a hematitic concretion. The left maxilla preserves the alveoli for the upper canine and all postcanine teeth. The anteriormost alveolus is large and would have housed the canine. Immediately posterior to it is a smaller, shallower pocket that must have held the roots to P1. However, this area is partly obscured by an encrusting layer of concretion, so precise details of the root morphology of P1 are unclear. The alveolus for P2 is an ovoid depression, elongated transversely. It is subdivided buccolingually by a septum, indicating that P2 was double-rooted, with subequal roots. P3-P4 are preserved in place and both are transversely elongated. P3 is oriented so that a line through its protocone and paracone is nearly perpendicular to the tooth row. P4 is oriented oblique to that line so that the protocone is positioned more mesially. The alveoli for M1, M2, and M3 (the lateral walls of the buccal pair of M3 alveoli are missing) indicate that these teeth each had three roots—a subequal pair of buccal roots and a much larger lingual root—and that these teeth declined in size from M1 through M3. M3 would have been approximately one half the maximum buccolingual width of M1.

The roof of the palate on the maxilla is nearly flat on its ventral (oral) surface. A rugose sutural surface for the opposing maxilla is present on the medial surface of the left and right maxillae anterior to P4, but this surface is missing posterior to this point. A groove lingual to the tooth row on the palatal surface extends posteriorly to the level of M2, paralleling the tooth row across its entire length. At least two circular depressions lingual to M1 and M2 are small palatine foramina and probably the position of the maxillary palatine contact. The left maxilla preserves a part of the floor of the nasal pharynx which forms an anteroposteriorly elongate trough-like shape.

On its lateral surface, the maxilla flares laterally to form the root of the zygomatic arch. The ventral portion of the arch begins at a position centered between M1 and M2. A portion of the ventral rim of the orbit is preserved, but the sutural contacts between the maxilla, zygoma, and lacrimal are not visible. The anterior rim of the orbit is significantly farther forward, over M1, in *Onychodectes*, *Huerfanodontorrejonius* , or *Alveugena* [24] and more posterior in *Psittacotherium* (over P3). The anterior rim appears to be over the space between P3 and P4 in *Schowalteria* [7] and over either P3 or P4 in *Stylinodon*. The placement of the anterior orbital rim is unknown in *Huerfanodonpolecatensis* and *Conoryctella*. A large and circular lacrimal foramen opens posteriorly from within the thin, rugose orbital rim. Dorsomedial to this, the preserved ragged edge of the maxilla may represent the sutural surface for the frontal. The infraorbital foramen is nearly circular and is positioned over P3. Schoch ( [2]: 49) stated that the foramen “appears” to be positioned above P3-P4 in the poorly preserved type. Therefore, the new material from New Mexico corroborates this interpretation and makes clear that the foramen is located in this position in *Wortmania*. The infraorbital foramen is over P3 in *Onychodectes*, *Conoryctes* (not over P4 as is indicated in the reconstruction in Schoch ( [2]: Figure 9), *Psittacotherium*, and *Ectoganus*, but over P2 in *Stylinodon*. The infraorbital foramen position is unknown for *Conoryctella* and *Huerfanodonpolecatensis* and we have not been able to determine the condition with certainty in *Huerfanodontorrejonius* . The infraorbital canal is nearly cylindrical.

A partial left squamosal (Figure 5E-I) preserves the posterior root of the zygomatic arch laterally, the lateral side of the braincase dorsomedially, and most of the glenoid fossa ventrally. The posterior surface is plastered with a large globule of hematite and therefore obscured. The posterior root of the zygomatic arch is narrow and nearly cylindrical in shape. The dorsal surface of the squamosal is relatively smooth and widely convex between the squamosal portion of the zygomatic arch and the lateral wall of the braincase. Posteriorly, the squamosal is bordered by the lateral portion of the lambdoidal crest. Ventrally, the glenoid fossa is shallowly concave, mediolaterally elongate, and ovoid in outline. The anteromedial part of the fossa is missing. A low ridge descends from the medial half of the posterior margin of the glenoid, forming the postglenoid process. The presence/absence of a postglenoid foramen cannot be determined. A protuberance, the mastoid process, largely obscured by hematite, is present posterolateral to the glenoid fossa and separated from it by the channel for the external auditory meatus. The mastoid process is similarly positioned, but larger, in *Psittacotherium* (NMMNH P-48436). It is more mediolaterally elongate in *Ectoganus* (see [25]) and *Stylinodon* (see [2,26]) and similarly extends posterolateral to the glenoid fossa. The mastoid process is positioned posterior to the glenoid fossa, medial to its lateral edge, in *Onychodectes* and *Conoryctes* [2]. The shape and position of the mastoid process is unknown in other taeniodonts.

A portion of the right basiocciptial (Figure 5J-K) preserves an occipital condyle and a portion of the margin of the foramen magnum, which is partly covered with a hematitic concretion. Flat and rugose surfaces are present lateral and dorsal to the occipital condyle. These surfaces, which intersect dorsally, are sutural contacts for the petromastoid and supraocciptial, respectively.

#### Mandible

The dentaries of NMMNH P-64001 (Figure 6) closely resemble those previously described by Schoch [2] based on several known specimens (AMNH 3394, AMNH 16342, UCMP 36528, and USNM 15428). As in all other dentaries of *Wortmania*, the symphysis of NMMNH P-64001 is large and unfused to the opposing symphysis, and the mandible is short and deep. The dentary is deepest at the symphysis and shallows posteriorly. The symphyseal region is ovoid and rugose, extending posteriorly to below p3. Details of this area are partly obscured in both dentaries by a concretionary film, but the symphysis on the left dentary (Figure 6D) is marked by an embayment on the posterior margin that marks the pit for what Patterson [12] and Schoch [2] interpreted to be the genioglossus muscle of the tongue. The anterior margin of the symphysis in both dentaries is missing, damage probably related to the displacement of both incisors.

Judging by the large size of the loose incisors, it is probable that only one pair of lower incisors was present as in derived “stylinodontids” (i.e., *Psittacotherium, Ectoganus*, and *Stylinodon*). The tooth row is short and posteriorly it curves lingually so that the alveoli of m3 are positioned adjacent to the lingual margin of the dentary and lingual to the anterobuccally extending flange that forms the anterior root of the ascending ramus. A small, semicircular mandibular angle (a posteroventral expansion of the dentary) extends posteroventrally from the horizontal ramus. Its posterior margin is transversely thickened. The condyle for articulation with the squamosal is positioned just above the level of the tooth row. It is large and transversely wide, centered on the plane formed by the mandible and angled so that its long axis projects dorsolaterally. Unfortunately, it is largely obscured by a hematitic concretion. The coronoid process is large, high, and sweeps posteriorly. The masseteric fossa on the lateral surface of the right coronoid process is shallow and demarcated anteriorly by a thin flange that is positioned approximately at the anterior margin of the coronoid process. Two mental foramina are visible on the lateral surface of the partial left dentary; the first is positioned about two thirds of the way up the mandible below p1 and the second is located about halfway up the mandible below p3. The dental foramen is not apparent, as it is evidently obscured by concretion.

#### Dentition

The dental formula of *Wortmania* is the subject of debate and has been reinterpreted several times since the initial description by Cope [9] of the type dentition (AMNH 3394). Cope [9] noted (p. 311) the “formidable teeth in front”, which presumably included the canines, and seven postcanine lower teeth. Cope [9] stated that only two upper teeth were preserved in place in the maxilla and that he was unable to find upper molars in the collection. Wortman [10] described the same specimen, identifying (p. 68) “…at least one pair of [upper] incisors and very probably two” as well as one or two pairs of lower incisors. Wortman [10] noted the large canines and identified three upper teeth in place: P3, P4, and M1. He found four lower premolars and four molars. Matthew [11] interpreted the dental formula of *Wortmania* to include a single pair of upper and lower incisors, a pair of upper and lower canines, three upper and four lower premolars and three upper and three lower molars (I1,C1,P3,M3/i1,c1,p4,m3). Patterson [12] indicated that *Wortmania* had four upper premolars, and stated that certain upper molars were then unknown for that taxon. Schoch [2] similarly reconstructed *Wortmania* as having one pair of upper and lower incisors, one pair of upper and lower canines, four upper and lower premolars, and three upper and lower molars (I1,C1,P4,M3/i1,c1,p4,m3), an interpretation with which we agree with and follow here based on NMMNH P-64001.

#### Incisors and canines

Specimen NMMNH P-64929 includes the crown of a lower canine and three fragments that likely represent portions of one or more canine roots (Figure 4). A smaller tooth with a single root that we tentatively identify as an upper incisor was found in close association with the canine crown and root fragments. The root of the upper incisor is nearly cylindrical, but is missing the root tip. The crown is preserved, but is highly worn through attrition. The preserved crown is circular in cross section as is the upper incisor of the type, AMNH 3394. A narrow band of enamel is preserved over about half of the tooth perimeter and this is interpreted to be the buccal tooth surface. The occlusal surface is smooth and as in AMNH 3394, is canted lingually about 45 degrees from the long axis of the tooth. This surface is nearly flat buccolingually but strongly convex in a mesiodistal direction. Wear has obliterated the original root enamel juncture for all but the buccal portion of the tooth and so we are unable to determine if the tooth enamel was originally restricted from the lingual tooth margin.

In NMMNH P-64001, the lower pair of incisors (Figure 7A-D) was found loose. What is tentatively interpreted to be the right lower incisor is cemented to the right dentary by a hematitic concretion (Figures 6A, 7A, D), which makes the lateral surface of the crown nearly inaccessible for study. Both incisors closely resemble those of *Psittacotherium* (see NMMNH P-44908) in being laterally compressed with a high labial peak, descending to a lower lingual buttress. The apex of the anterior peak and platform are devoid of enamel. However, unlike *Psittacotherium*, the enamel of the tooth extends completely around the tooth’s perimeter rather than being restricted to just the labial half of the tooth (although see below). This *Psittacotherium*-like condition has been described for *Wortmania* previously [10]. The enamel extends farther basally on the labial side of the incisor and with continued wear, the enamel on the posterior half of the tooth would be obliterated as is found in the type, AMNH 3394 [10]. The condition is different in the lower incisors of *Psittacotherium* (preserved with NMMNH P-44908), in that the typical enamel ends abruptly on both the mesial and distal sides of the tooth near the median. A small area of enamel near the base of the crown is a bit irregular, bearing small pits and grooves where the enamel is thinner. Lingual and adjacent to the abrupt edge of enamel, along a little worn or unworn strip at the base of the crown, is an area of high reflectance (shine), but of the same color as the exposed pulp cavity. This small strip may represent the presence of a very thin layer of enamel.

Both the left and right lower canines of NMMNH P-64001 are preserved in place (Figure 7E-H). The right canine is damaged and a fragment of the crown is missing, apparently because it was spalled off of the tooth prior to burial. The apex of the tooth is partly obscured by a globule of hematite. The left canine is well preserved. The roots are massive, bulbous, and club-like, and extend basally to below the p1. The crown preserves enamel only the labial surface of the tooth. The enamel extends farther basally on the distal side of the tooth than on the mesial side. The tooth is worn so that the lingual surface is nearly flat near the apex, producing a chisel-like apex. The tooth expands basally, especially in the lingual direction, creating a rounded platform or buttress (sensu [12]) near the base of the crown. The enamel is smooth over most of the preserved labial surface, but near the enamel root juncture the external enamel surface is irregular due to the presence of a few apicobasal ridges.

A loose upper left incisor of NMMNH P-64001 (Figure 8D-F) is complete except for a portion of the root. The root is partly covered by a concretion, but the crown is unobscured. The tooth is ovoid in cross section and elongated anteroposteriorly, unlike the more nearly circular cross sectional shape observed for the upper incisor preserved with AMNH 3394 or the tooth tentatively identified as the upper incisor in NMMNH P-64929. The enamel is smooth, and extends farther basally on the anterior (labial) surface of the tooth. The apex of the tooth is beveled to a flat, horizontal wear facet. The apical facet is confluent with a posterior and medial wear facet that extends to the base of the crown, forming a wear band that completely prevents the enamel from encircling the tooth. It cannot be determined if the enamel was originally continuous around the circumference of the tooth.

A single left upper canine of NMMNH P-64001 was found loose and is complete except for the apex (Figure 8A-C). It is partly covered with a hematitic concretion which obscures much of the root and the lingual, enamel-free portion of the crown. As with the lower canines, the root of the upper canine is massive and bulbous. Additionally in common with the lower canines, the enamel extends more basally on the distal side of the tooth. The canine is nearly circular in cross section at the base of the crown. Most of the crown is covered with enamel except for a apicobasal strip on the lingual side of the tooth. The enamel on the mesial side of the tooth adjacent to this strip is deeply pitted with apicobasal grooves that extend deeply into the enamel, and in places punch through the enamel completely, suggesting that this area of the tooth is at or near the original edge of enamel. The distal margin of the enamel-less band on the lingual face of the tooth is nearly completely obscured by concretion, but some relatively deep apicobasal grooves are present in the enamel, parallel to the lingual margin of enamel.

These features indicate that in *Wortmania*, the lack of enamel on the lingual face of at least the upper canines was due not solely to wear, but to the lack of enamel development as in the derived stylinodontids *Psittacotherium*, *Ectoganus*, and *Stylinodon* [2,10,11]. This morphology is different than that seen in *Conoryctes*, best observable in NMMNH P-61799, in which the enamel of the upper canine is preserved in two bands: a mesiodistally narrow band on the labial surface of the tooth and a thinner band on the lingual side of the tooth. The space between the bands is marked by flat wear surfaces on both the labial and lingual sides of the tooth. *Conoryctes*, *Conoryctella* (see [2]), and other “non-stylinodontid” taeniodonts for which lower canines are known, do not develop a basal posterior buttress, although Fox and Naylor [7] described the presence of a (p. 400) “robust buttress” of the lower canines below a deep posterior wear facet on the upper canines of *Schowalteria*. Unlike the lower canines, the enamel surface of the upper canine is not smooth as in “non-stylinodontid” taxa, but rugose, with a pattern of small shallow pits and low apicobasally oriented grooves

#### Premolars

The lower premolars are best seen in place in the right dentary of NMMNH P-64001, but p1, p3, and p4 are all partly obscured by concretion (Figure 7E-K). The first three premolars are or were all positioned oblique to the long axis of the jaw, whereas the fourth premolar is in line with the jaw axis. The first three premolars are similar in morphology to each other, as they all possess a mesial dominant bulbous cusp followed by a distolingually placed heel. The fourth premolar, on the other hand, possesses a mesial cingulid that appears to represent a paracristid, but details of its apex are obscured by concretion. A right loose premolar is tentatively identified as a p2. It is well-preserved and ovoid in cross section with a dominant and bulbous anterior cusp, the protoconid, and a low posteriorly heel with a single low cuspid. The enamel is smooth with the enamel extending more basally on the buccal side of the tooth than on the labial side. The apex of the primary cusp curves distally (considering that the tooth would be in the dentary at an oblique angle when articulated in its alveolar socket). The apex of the crown is beveled by horizontal occlusal wear, exposing a small circle of dentine, and flat wear facets are present on the mesial and distal surfaces of the crown, adjacent to the apex. Similar wear facets are present on the right p2 and left p3-p4 (the apices of both left and right p1s are missing), but in those teeth the mesial wear facet has breached the enamel, exposing thin strips of dentine on the mesial and distal faces of the primary cusp.

A loose, small, single-rooted, and poorly preserved tooth of NMMNH P-64001 is interpreted to represent a P1 (Figure 8I-J). It has a large bulbous primary cusp and a distal cingulid. Two loose premolars are the left and right P2s. The right P2 is well preserved with a large single root, a large bulbous primary cusp (the paracone), and a lingual heel supporting a single small, median eminence (the protocone). A wear facet is developed on the apex and continues as a strip to the base of the tooth mesially (Figure 8H-J). Both left and right P3 and P4 are preserved. The right P3-P4 of P-64001 are loose (Figure 8I-J) and the left P3-P4 are in place in the maxilla (Figure 8G-H). The right P3 and P4 have three roots. Based on the alveoli and teeth preserved in place, the P2-P3 were both positioned so that the long axes of the crowns were oriented perpendicular to the cheek tooth row. P4 is oblique to the tooth row with the protocone positioned more mesially than the paracone. P3 and P4 are bulbous with high paracones and lower, smaller, nearly circular protocones. The P3 lacks a parastyle and has a small, distal metastyle near the base of the paracone. It lacks conules and basal cingulae. The P4 parastyle is present as a low basal cusp on the mesiobuccal corner of the tooth. A short basal cingulid continues from the paratyle for a short distance on the buccal side of the tooth. A metastyle is similar in size to the parastyle and present at the base of the tooth distal to the paracone. There is no evidence for a metacone, but the paracone is mesiodistally elongate and a wear facet is developed as a strip along the distal edge of the paracone, extending to the metastyle, possibly erasing evidence of its presence.

#### Molars

The lower molars of NMMNH P-64001 are represented by a right m1 in place (Figure 7I-K), a fragmented loose right m2, a loose left m1, and a loose left m2 (Figure 7L-P). All preserved lower molars have two roots. The trigonids are subequal in width and length to the talonids, but are higher. The occlusal surfaces of the teeth are highly worn, resulting in a single, nearly horizontal wear facet on each tooth. A cleft on the lingual margin of m1, mesial to a narrow entocristid, marks the opening of the talonid basin at the distal base of the metaconid.

Upper molars of NMMNH P-64001 are represented by loose left and right M1s and M2s (Figure 8I-K). The left M2 is cemented to the left dentary and mostly obscured by a hematitic concretion (Figure 6D). The left and right M1 (Figure 8F-G) are both partly obscured by hematite. The right M1 has a globule of hematite adhering to the occlusal surface near the median of the tooth, but it is confined to the occlusal wear facet and doesn’t appear to be obscuring any significant morphological features. A lingual portion of the right M1 protocone is missing. The left M1 is less well preserved because a thin layer of hematite partly obscures some features of the crown and the parastyle is missing. The right M2 is highly worn by occlusal wear, but is otherwise well preserved. All upper molars possess three roots: two small and subequal buccal roots and a larger root under the protocone. Based on the alveoli preserved in the left maxilla (Figure 8H), the molars decrease in size from M1 to M3. M3 was evidently much smaller than M1 and asymmetrical with a reduced distal part of the tooth.

The M1 (Figure 8I-K) is transversely wide with a large protocone, whereas the paracone and metacone are much smaller, closely appressed, and merged together at their bases. The paracone is larger than the metacone. A weak preparacrista extends from the preserved apex to the base of the tooth. A deep cleft separates the paracone from the parastyle. The parastyle consists of a prominent ridge oriented transversely and subsiding distobuccally to a low and weak, cuspidate ectocingulum. Lingually, the parastyle appears to be confluent with a preparaconule *crista*, but occlusal wear has obliterated most of the original external surface from this area of the tooth. Distally, a low metacrista extends from the apex of the metacone and curves buccally to extend to the distobuccal corner of the tooth. The metastyle is present as a thickening at the intersection of the metacrista and ectocingulum, but does not form a separate cusp.

The M2 (Figure 8I-K) resembles M1, but is smaller and more asymmetrical, with a more reduced distal half and relatively smaller metacone. The preparacrista is stronger and the parastyle is more prominent and more buccally positioned than in M1. Also compared to M1, the ectocingulum of M2 is less well defined and not continuous between the para- and metastyles. On both the M1 and M2, a smooth and transversely concave wear facet is present over the protocone and presumably the conular surface, and extends down the lingual face of the paracone. The para- and metaconules are missing, presumably removed by occlusal wear.

Patterson [12] described the basal extension of the enamel on the lingual side of upper teeth and on the buccal side of lower teeth as related to a “rolling eruption” that results in the course of eruption and attritional wear. While buccal extension of the upper premolars of *Wortmania* is clearly evident based on NMMNH P-64001, the upper and lower molars do not preserve the substantial amounts of lingual distension of enamel (upper molars; Figure 8K) or buccal distention of enamel (lower molars; Figure 7N) that is seen in some other Paleocene taeniodonts such as *Conoryctes*, *Huerfanodon*, and *Psittacotherium*.

#### Ulna

**Figure 13:**
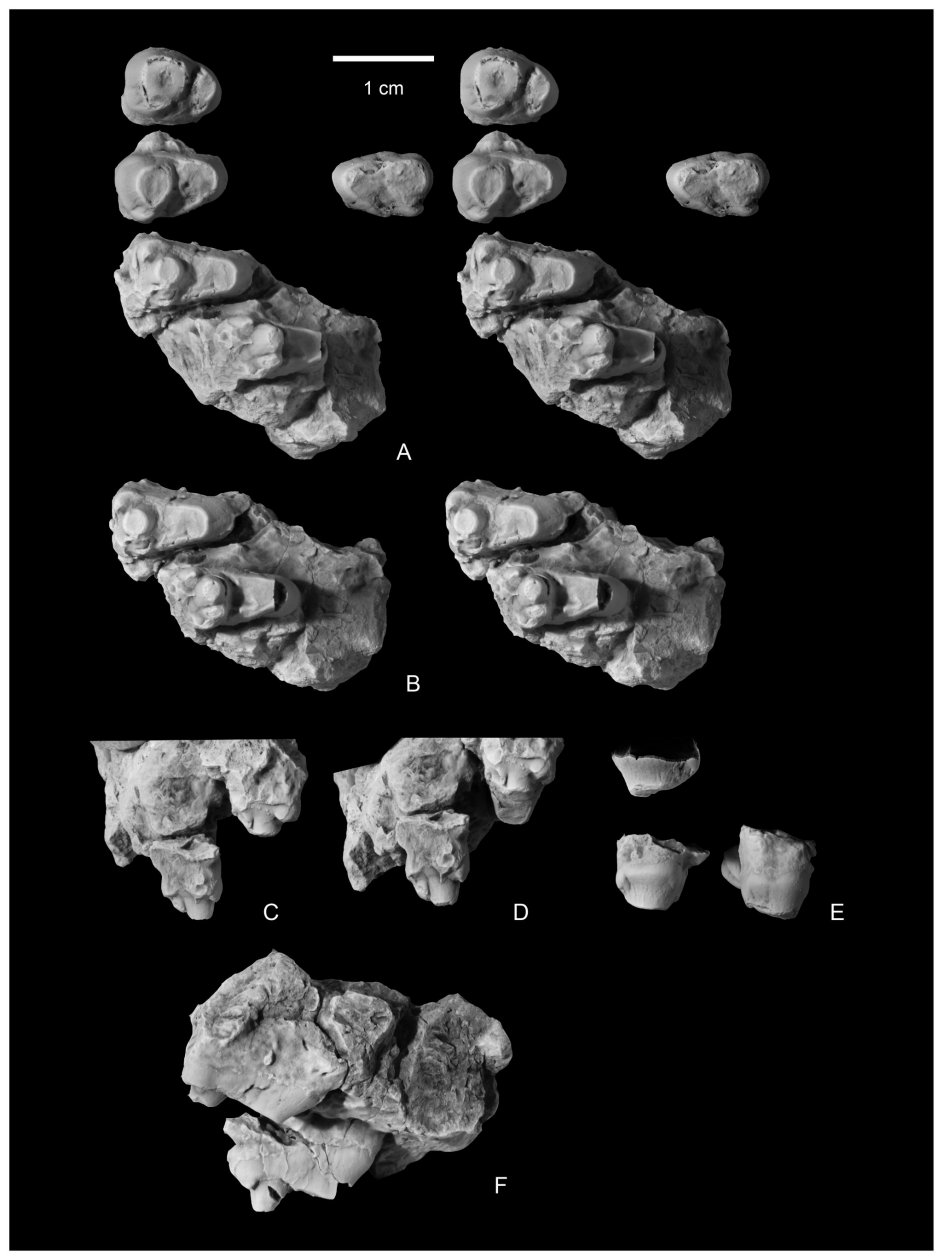
Holotype of *Robertschochiasullivani* (NMMNH P-9000, here argued to be a subjective junior synonym of *Wortmaniaotariidens* ), right P3-M2, left P4 in occlusal view (A; stereo pair; M1 in occlusal [upper] and M2 in occlusal [lower]); C, buccal view of M1; D, buccal view of M2; E, buccal views of premolars (left P4 [upper]; right P3, P4 [lower]); F, mesial view of M1 and M2.

Specimen NMMNH P-19460 includes both left and right ulnae, each of which is more than 50 percent complete. Both, however, are missing much of the olecranon processes and a portion of the distal end of the shaft. The right ulna is more complete than the left, and it preserves part of the distal end. Both ulnae are similar to the partial ulna of the type of *Wortmaniaotariidens* (AMNH 3394; Figure 3B), which is figured by Matthew ( [11]: Plate lxii, Figure 2) and Schoch ( [2]: Figure 13). The right ulna of the new specimen, NMMNH P-19460 (Figure 9), is more complete and better preserved than that of the type, which allows for new anatomical observations.

The ulna is a robust element that is mediolaterally compressed and anteroposteriorly deep. It has a sigmoidal outline in lateral view with a ventrally convex ventral margin over the distal half of the bone and a posteriorly concave posterior margin over the distal half. This sigmoidal outline is not apparent on the less complete left ulna or on the partial ulna of the type (AMNH 3394; Figure 3B) because these bones are broken proximal to the concave posterior margin of the distal half. Similarly, the best preserved ulna of *Onychodectes* (AMNH 16410; see [2]: Plate 7, Figures 2-3) is broken distally, precluding the determination of whether it had a posteriorly concave posterior margin. Matthew [11] described this specimen as complete, but it has been broken since and Matthew’s description does not note whether the distal portion of the bone was straight or sigmoidal [2]. All known ulnae of *Ectoganus* are also broken distally [2], but *Conoryctella*, (NMMNH P-25056), *Conoryctes* (NMMNH P-48052), *Psittacotherium* (AMNH 2453), and *Stylinodon* (Schoch [2]: Figure 36; YPM 11096) clearly possesses ulnae with the sigmoidal curvature similar to what is seen in *Wortmania*.

The semilunar (trochlear) notch, for articulation with the distal end of the humerus, is semicircular in lateral view. This is also the case in the type of *Wortmania* (AMNH 3394) and *Psittacotherium* (Schoch [2]: Plate 30; NMMNH P-56951). In contrast, the notch is more of a shallow, half ovoid shaped concavity in *Ectoganus* (Schoch [2]: pls. 34, 46) and *Stylinodon* (Schoch [2]: Figure 36; YPM 11096). In the new specimen of *Wortmania* the anterior surface of the semilunar notch is saddle-shaped, with a shallow mediolateral convexity, as is present in other taeniodonts. The facet for the radial head is located medial to the coronoid process, on the anterior surface of the ulna. It is separated from the trochlear surface of the semilunar notch by a narrow rugose band. The radial head facet is nearly flat, smooth, and ovoid in anterior view and faces anteriorly and slightly medially. This is similar to what is seen in other taeniodonts (i.e., *Onychodectes, Conoryctella, Conoryctes, Ectoganus, Stylinodon*) and in contrast to the ulnae of several other “archaic” Paleogene mammals, among them *Ectoconusditrigonus* , *Claenodon* *ferox* , and *Periptychuscarinidens* , which have a more laterally directed and more deeply concave facet for the radial head ( [11]; personal observation).

The lateral face of the ulna bears a shallow longitudinal depression over its preserved length. Several small foramina open from near the midpoint of the groove below the semilunar notch. The partial ulna that is part of the type of *Onychodectes* *rarus* (AMNH 3405) bears a similar groove and foramina in the same location. The longitudinal depression may represent the origin of the abductor pollicis longus (after [27]). In *Wortmania*, the depression is anteroposteriorly broad. A similar depression is anteroposteriorly narrower and confined to the anterior part of the lateral face of the ulna in other Paleogene mammals including *Arctocyonprimaevus* [27], *Ectoconus*, *Periptychus*, *Claenodon* (personal observation), a variety of other early placental mammals including some mesonychians [28], and some taeniodonts including *Conocryctella* (NMMNH P-25056) and *Conoryctes* (NMMNH P-48052). *Psittacotherium* possesses only a shallow depression distal to the semilunar notch and the anterior portion of the lateral ulnar surface becomes convex distally. However, a distinct a longitudinally elongate ovoid concavity is restricted to the posterior and distal part of the lateral surface. A weak ridge demarcates an ovoid region in approximately the same location in *Wortmania*, but this area is not strongly concave or distinct.

The medial face of the ulna has an anteroposteriorly narrow groove over its proximal half, probably representing the proximal fossa for the anconeus muscle. This is similar to what is found in other taeniodonts including *Onychodectes* (AMNH 3405, 16410), *Conocryctella* (NMMNH P-25056), *Conoryctes* (NMMNH P-48052), and *Psittacotherium* (AMNH 2453, NMMNH P-56951). The distal half of the ulna is anteroposteriorly convex from the distal base of the coronoid process. A low ridge descends from the medial edge of the coronoid process and continues toward the midpoint of the lateral surface. A second ridge bearing a rugose muscle attachment scar originates near the midshaft of the ulna, medial to the margin for the attachment of the interosseous ligament at the anterior edge of the bone. It converges on the first ridge toward the midpoint of the lateral face of the ulna near the distal end of the bone.

The ventral margin of the ulna is mediolaterally narrow distally but expands in width proximally to form a shelf with a sharp lateral edge, which begins below the coronoid semilunar notch. This ventral shelf continues to expand laterally towards the olecranon process.

Dorsally, the ulnar shaft is relatively flat distal to the semilunar notch and the facet for the radial head, but it narrows in mediolateral width distally into a uniform, transversely convex ridge that arches proximodistally. A deep, proximodistally elongate fossa on the margin of the dorsolateral surface, just distal to the coronoid process, presumably marks the insertion for the m. *biceps brachii* and m. *brachialis* [15]. A similar fossa is present in various other Cretaceous and Paleogene mammals including *Ukhaatherium* [29], *Arctocyon* (see 27), *Escavadodon* [30], *Claenodon* (personal observation), *Periptychus* (personal observation), and *Ectoconus* ( [11]; personal observation). The dorsal surface of the ridge is rugose for the interosseous membrane toward its distal end.

A fragment of the distal end of the right ulna preserves the distal articular surface (Figure 9A). The styloid process takes the form of a low ovoid process that forms a distally convex platform over the distal half of the distal ulna. This process forms the condyloid articular surface for articulation with the cuneiform. The distal end of the ulna is known for two other taeniodont taxa: *Psittacotherium* (based on AMNH 2453 and NMMNH P-47687) and *Stylinodon* (YPM 11096 [2]: Plate 55, Figures 5-6). Both of these taxa possess a relatively much larger and more distally extending, finger-like projection of the styloid process.

#### Radius

Both left and right radii are preserved as part of NMMNH P-19460 (Figure 10). The right radius is nearly complete and the left radius is essentially complete, as it is only missing a slight portion of the distal end. Both bones show damage to the proximal end, mostly confined to parts of the margin of the radial head. The distal end of the right radius is also damaged so that much of the distal surface and perimeter is missing. A portion of the distal end of the right radius is preserved, but is free.

The shaft of the radius is bowed in lateral and medial views. As with the sigmoidal curvature of the ulna, this bowing is only clearly apparent on the more complete left radius, and is not clearly recognizable on the less complete right specimen that is missing most of the distal end. The bowing is also clearly evident on the left radius of the type specimen (AMNH 3394, Figure 3A), but that specimen is missing the distal end. The radius is similarly bowed in *Psittacotherium* based on AMNH 2453 (see Wortman [10]: Figure 9). We are unsure of the condition in *Ectoganus* and *Stylinodon*. In the new *Wortmania* specimen, the proximal half of the shaft is nearly cylindrical, expanding abruptly in the mediolateral and anteroposterior directions is it approaches the proximal end. The radial head is ovoid, with a concave proximal surface surrounded by a narrow “lip” laterally for articulation with the trochlea of the humerus. The ulnar side (this is equivalent to the posterior side of the ulna as this bone was habitually pronated) of the radial head of the radius supports an ovoid facet for articulation with the nearly flat, complementary facet for the radial head of the radius on the ulna. The lateral margin of this facet is not preserved on either the left or right radius. The preserved portion of the rim of the radial head is smooth and only shallowly convex in a transverse direction. Distal to the rim of the radial head, and separated from it by only a narrow notch, the bicipital tuberosity is a prominent, ovoid, and rugose protuberance.

Distal to midshaft, the radius is supports a prominent longitudinal crest on its dorsolateral surface. The opposite side of the shaft is scarred by a rugose attachment surface for a strong interosseus membrane that extends over the distal two thirds of the shaft.

The distal ends of both radii are incomplete, but both preserve a portion of the distal articular surface and the styloid process. The styloid process is represented by two low knobs that extend distally from the margin of the distal surface. The distal surface itself is incompletely preserved. A portion of the distal end of the left radius preserves the styloid process and the adjacent portion of the distal surface, including a small portion not preserved on the right radius. Together, these form a nearly complete distal surface, permitting observation of this important region. This specimen indicates that the distal surface formed a single smoothly flat to shallowly concave depression for articulation with the lunar and scaphoid.

#### Manus

**Figure 14:**
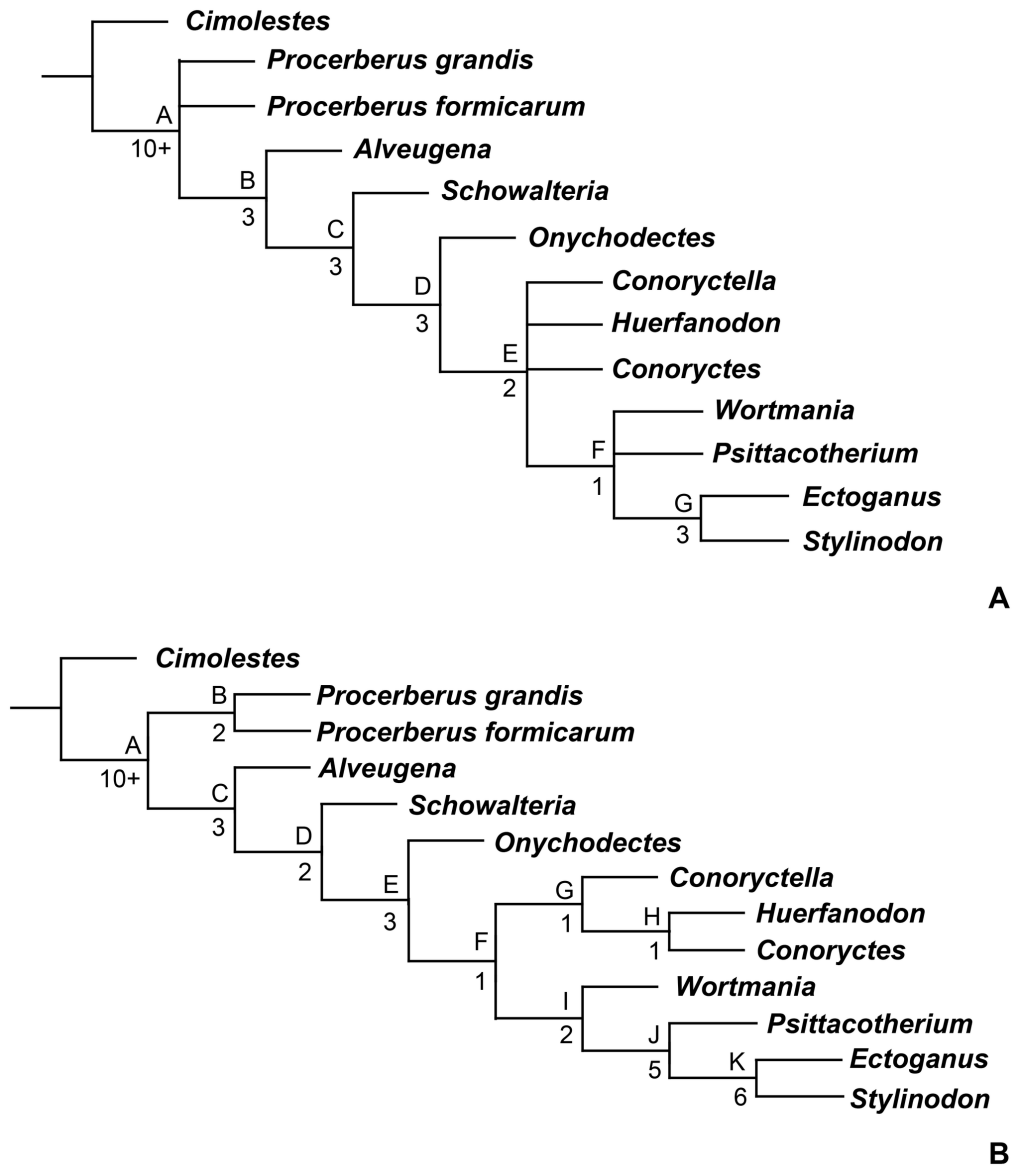
Results of a phylogenetic analysis based on a modified version of Rook and Hunter’s [8] character matrix incorporating evidence supplied by new *Wortmania* (= *Robertschochia*) specimens and other changes (see text; Appendices S1 and S2). A, strict consensus of six most parsimonious trees resulting from running all multistate characters unordered (Consistency Index = 0.648, Retention Index = 0.667); B, strict consensus of two most parsimonious trees resulting from running multistate characters as ordered ( Consistency Index = 0.562, Retention Index = 0.679) (see text for details). Numbers below left of nodes are Bremer branch supports calculated from a pool of up to 50,000 suboptimal trees of up to 10 steps longer than the shortest trees obtained. Letters above each node in the cladogram refer to nodes in the diagnoses in Appendices S3 and S4.

Only three bones of the manus of *Wortmania* have previously been described: a possible left lunar, a right second metarcarpal (Schoch’s [2] identification of this bone was tentative, but we confirm it is a metacarpal II), and an ungual (see Schoch [2]: Figure 14, Plate 20), all based on the type specimen (AMNH 3394; Figure 3C).

The new specimen NMMNH P-19460 includes several manual elements, including several not previously described. Among these are a bone tentatively identified as a partial left magnum, left and right metacarpals II – IV, and fragments of two manual unguals (Figures 11-12).

#### Magnum

A partial bone is tentatively identified as a left magnum (Figure 11A). This bone can be compared with those of the partial manus of *Psittacotherium* based on AMNH 2453 (Schoch [2]: Figure 19, Plate 30, Figure 1; Wortman [10]: Figures 12-13; Matthew [11]: Figure 66, Plate lxi, Figure 2) which preserves the lunate, cuneiform, partial unciform, and partial magnum. Unfotunately, the manus of AMNH 2453 is now set in plaster, hampering comparison. The new *Wortmania* specimen possesses a plantar tubercle and is missing its dorsal portion. The distal surface has a good fit with the proximal end of the left metacarpal III. The proximal side bears two articular surfaces; both are concave and divided by a dorsoventrally aligned ridge. Based on comparison to *Psittacotherium* (AMNH 2453), the medial (and larger) of the two proximal articular surfaces articulated with the trapezoid. The lateral of the proximal surfaces articulated with the lunate. The medial surface of the magnum articulated with the trapezium and the lateral surface articulated with the unciform.

Contact between both the centrale and lunar on the proximal surface of the magnum is also present in *Onychodectes*, *Psittacotherium*, and *Ectoganus*, but in *Stylinodon* this surface has been described as contacting the lunar only ( [2]: Figure 38). However, Schoch’s illustration shows a small concave notch on the medial side of the proximal surface of the magnum and this notch may articulate with the centrale, which is unfortunately unknown for this specimen of *Stylinodon*. A more complete specimen of the *Stylinodon* manus is described and figured by Turnbull ( [26]: Figure 16). He describes the magnum as articulating with the lunar proximally and does not mention any contact with the centrale. Figures of the specimen, however, do indicate that the proximomedial corner of the magnum likely made contact with the centrale, although this articular facet is not as large as in *Onychodectes*, *Psittacotherium*, and the new specimen of *Wortmania*, in which this facet is a broad and concave margin that occupies nearly half of the proximal surface.

The morphology of taeniodont magnums gives some potential insight into some features of the lunate, which is important because this latter carpal bone is not preserved in the new specimen. The distal surfaces of the lunate of another specimen of *Wortmania* (AMNH 3394) and *Psittacotherium* (AMNH 2453) have a deeply-excavated, oblong, cup-shaped concavity for receiving the head of the magnum [10]. A similar condition is seen in the carpal bones of *Onychodectes* preserved with the partial manus of AMNH 16528 (see [2]: Figures 5 and [11]). In this specimen, the proximal surface of the magnum bears a prominent, hemispherical protuberance that fits into the receiving cup-shaped surface of the lunate [2]. The new magnum fragment of NMMNH P-19460 that we describe here does not preserve a protuberance on its proximal surface, but we attribute this to the incompleteness of the bone and hypothesize that it would have been present if the specimen were complete.

#### Metacarpals

The metacarpal bones are all short and stout. Metacarpal III (Figure 11C, F) is the largest, proximodistally longest, and mediolaterally widest of the three metacarpals (Table 2). Metacarpal II (Figure 11B, E) is slightly narrower in width and much shorter proximodistally than metacarpal III. Metacarpal IV (Figure 11D, G) is subequal in length to metacarpal II, but with a more slender and gracile shaft and distal end.

#### Metacarpal II

Metacarpal II (Figures 11B, E, 12) is nearly rectangular in dorsal view, as it expands only slightly mediolaterally at its proximal and distal ends. The shaft is elliptical in cross section at midshaft, but expands mediolaterally and ventrally towards the distal surface, and in a ventral direction towards the proximal end of the bone. The dorsal surface bears a prominent extensor tubercle on the lateral side, about one third of the way along the shaft. A similar, prominent extensor tubercle is found in *Onychodectes* (AMNH 16528; personal observation), and *Stylinodon* (YPM 11096). A depression on the medial side of the dorsal surface, near the margin of the distal condyle, marks an extensor fossa, which is absent in *Onychodectes* and *Conoryctes* but cannot be determined for other taeniodont taxa.

The proximal articular surface, for articulation with the trapezoid, is approximately rectangular in outline in proximal view, with a nearly straight medial border and a more complex and sinuous lateral border for articulation with metacarpal III. The proximal surface is shallowly convex in a dorsoventral direction, with a nearly median dorsoventrally aligned groove. The medial edge of the proximal surface is sharp and the lateral edge forms a smoothly rounded lip. In medial view, a narrow smooth band along the proximal edge of the shaft marks the articular surface for the trapezoid or possibly for metacarpal I. Laterally, the articular surface for metacarpal III is cup-shaped and overhangs the proximal end of metacarpal II.

The distal articular surface has a strongly convex dorsal margin, but the ventral half has a flat profile and is oriented nearly 45 degrees from the vertical when the metacarpals are in articulation. A strong median ventral keel is confined to the ventral half of the distal articular surface. In distal view, the distal articular surface is asymmetrical, with the dorsal surface decreasing in height medially. The medial and lateral sides of the distal end each bear a deep dorsoventrally elongate pit for the collateral ligaments.

The second metacarpal of *Wortmania* (Figures 3C, 11B, E, 12) closely resembles the corresponding bone in both *Psittacotherium* ( [2]: Figure 19; NMMNH P-47687) and *Stylinodon* ( [26]: Figure 16). In all three taxa this metacarpal is proportionally short and stout, although in *Psittacotherium* and *Stylinodon* it is slightly more robust and proportionally stouter than in *Wortmania*. This can be quantified with a ratio of proximodistal length divided by mediolateral width at midshaft, which shows *Wortmania* (2.88) to have a proportionally longer metacarpal II than the aforementioned specimens of *Psittacotherium* (1.88) and *Stylinodon* (1.83). *Stylinodon* also shares with *Wortmania* the sinuous lateral edge of the proximal surface for articulation with metacarpal III ( [26]: Figure 18). *Onychodectes*, on the other hand, has a metacarpal II that differs strikingly in morphology with that of *Wortmania* (and by extension *Psittacotherium* and *Stylinodon*). The length:width ratio of one well preserved specimen (AMNH 16528) is 7.0 ( [2]: Figure 5), meaning that this bone is proportionally much longer and more gracile in *Onychodectes* than in *Wortmania*. Furthermore, *Onychodectes* has a broadly concave lateral margin of the proximal surface in proximal view, not the sinuous shape of *Wortmania* and *Stylinodon*, and has a more pronounced mediolateral expansion at its distal end than in *Wortmania*, *Psittacotherium*, and *Stylinodon*.

#### Metacarpal III

In dorsal view, the nearly rectangular shaft of metacarpal III (Figures 11C, F, 12) is similar to that of metacarpal II. It bears a knob-like extensor tubercle on its medial side, about one quarter of the way down the shaft. Proximally, a thick, laterally-projecting prong extends to articulate with the unciform and to separate the magnum from metacarpal IV.

Proximally, most of the articular surface contacted the magnum over a rectangular area that is dorsoventrally elongate and saddle-shaped; the saddle is shallowly convex dorsoventrally and more strongly concave in a transverse direction. The medial edge of the proximal articular surface articulated with the distolateral edge of the trapezoid and the lateral edge has a dorsoventrally elongate surface for articulation with the unciform. The medial surface of metacarpal III (see Figure 11G) bears a narrow lip for articulation with the overhanging facet on metacarpal II. Laterally, adjacent to the facet for the unciform is a separate narrow band that is distolaterally concave. This is an overhanging contact surface for metacarpal IV. Both the lateral and medial edges of the proximal surface are concave in proximal view.

The distal surface resembles that of metacarpal II, but it is symmetrical and rectangular in distal view, unlike the asymmetrical shape of metacarpal II (and metacarpal IV, below). As in metacarpals II and IV (below), the long axis of the distal surface it is oriented perpendicular to the shaft in dorsal view. In distal view, the dorsal margin of the distal surface is convex whereas the ventral surface is nearly flat, and oriented at about 45 degrees from the vertical when the metacarpals are in articulation. In lateral or medial view, the distal edge of the median keel is approximately parallel to the medial and lateral edges of the distal articular surface. The keel is brought into relief by grooves on either side, which are impressed into the distal articular surface.

As is the case with metacarpal II, the short and stout metacarpal III of *Wortmania* more closely resembles the corresponding bone in *Psittacotherium* ( [2]: Figure 19) and *Stylinodon* ( [2]: Figure 38 [26]: Figure 16) than the proportionally longer and more gracile element of *Onychodectes* ( [2]: Figure 5). *Onychodectes* also has a wider mediolateral expansion distally than *Wortmania*, *Psittacotherium*, and *Stylinodon*. In *Stylinodon* the lateral edge of the surface is slightly concave, but much less so than in *Wortmania*, and the medial surface is flat ( [26]: Figure 18).

#### Metacarpal IV

The shaft of metacarpal IV (Figures 11D, G, 12) is proportionally more gracile, and is more nearly circular in cross section at midshaft, than those of metacarpals II and III. Furthermore, the proximal end is relatively more expanded relative to the shaft in metacarpal IV than in metacarpals II-III.

The proximal end of metacarpal IV resembles an equilateral triangle in proximal view, with the apex pointed ventrally. The proximal surface is generally broadly rounded both transversely and dorsoventrally, with a median, shallow, and narrow dorsoventrally-aligned groove. A median lip along the medial margin of the proximal articular surface is the contact surface for metacarpal III. Laterally, the proximal end of metacarpal IV projects proximolaterally and bears a relatively small, nearly square contact surface for metacarpal V. It is shallowly concave in a dorsoventral direction. The proximal articular surface makes contact with only the unciform, which is also the case in other taeniodonts (e.g., Onychodectes, Psittacotherium [2]: Figure 19, Stylinodon [2]: Figure 38, [26]: Figure 16).

The distal end of metacarpal IV is similar to those of metacarpals II and III, but is more rounded in dorsal view, compared to the relatively flat distal margins of metacarpals II and III. In distal view, the articular surface is strongly asymmetrical and decreases markedly in height laterally. As with metacarpals II and III, the posterior portion of the distal articular surface is marked by a pronounced dorsoventrally-oriented ridge at its center, which is flanked by deep grooves.

As with metacarpals II and III, the metacarpal IV of *Wortmania* is more similar in its robust proportions to those of *Psittacotherium* and *Stylinodon*, and differs from the gracile metacarpal IV of *Onychodectes*. Furthermore, in *Wortmania*, *Psittacotherium*, and *Stylinodon* metacarpal IV is noticeably more slender in its midshaft width than metacarpals II-III, but this is not the case in *Onychodectes* in which the three central metacarpals are of the same approximate midshaft width ( [2]: Figure 5). The shape of the distal articular margin in dorsal view in *Wortmania*, which is substantially more rounded compared to the flat surfaces of metacarpals II-III, is distinct from that of other taeniodonts. *Onychodectes, Psittacotherium* and *Stylinodon* possesses a flat distal surface when metacarpal IV is seen in dorsal view, similar to the flat surfaces of metacarpals II-III ( [2]: Figures 5, 19, 28, [26]: Figure 16).

#### Manual Unguals

Specimen NMMNH P-19460 includes fragments of two unguals consist of part or most of the proximal end (Figure 11K-L). A small fragment may represent a small portion of the proximal end of a third ungual. They both are portions of large, mediolaterally compressed, and deep unguals like those found in the type of *Wortmaniaotariidens* (Figure 3D) and other stylinodontid taeniondonts (*Psittacotherium*, *Ectoganus*, *Stylinodon*), but differing from the mediolaterally broader unguals of *Onychodectes* ( [2]: Figure 5). The more complete ungual preserves most of the proximal end, but is missing the dorsal edge. The less complete ungual includes a portion of the proximal end, but includes only the ventral portion of the bone. The more complete ungual reveals a nearly ovoid outline in proximal view, differing from the more triangular outline of *Onychodectes*. The proximal surface is subdivided into two subequal dorsoventrally-elongated concave surfaces divided by a median ridge. The ventral surface of both fragments is nearly flat, but a portion of the plantar tubercle is preserved. The dorsal margin of the ungual was strongly convex in a transverse direction.

## Discussion

### Preservation of the New Specimen and the *Wortmania* Type

The preservation of NMMNH P-64001, characterized by the distinctive gray-green color with adhering globules of hematite, is typical for fossils from the De-na-zin Wash area and is extremely similar to of the type of *Wortmaniaotariidens* (AMNH 3394; Figures 1-3). D. Baldwin collected the type specimen in 1885 from what Cope [22] originally reported to be the “Puerco Formation,” San Juan Basin, New Mexico but a more precise locality was evidently not given by Baldwin. The Puerco Formation sensu Cope [22] is equivalent to what is now termed the Nacimiento Formation (see [31]). Wortman [10], leader of the first two American Museum of Natural History expeditions to collect Paleocene mammals from the San Juan Basin [13,32], indicated that *Wortmania* (=*Hemiganus*) was from the “Lower Puerco,” beds now regarded to be Puercan in age [31]. This strongly suggests that the type was also collected from “upper fossil level of Puerco” or “*Polymastodon* horizon” (sensu [33]) and equivalent to “fossil zone B” of Williamson [13]. Thus, NMMNH P-64001 may represent a topotype, although this is not possible to know for certain.

### Dental Morphology and Its Implications for Taeniodont Alpha-Level Taxonomy

The new specimen NMMNH P-64001 is the dentally most complete specimen of *Wortmaniaotariidens* . The dentary, and the canine, lower incisors, and premolars are nearly identical to those of the type of *W* *. otariidens* , AMNH 3394. In addition, the new specimen provides, for the first time, an associated and relatively complete upper and lower dentition.

Lucas and Williamson [6] named the taeniodont *Schochiasullivani* (*Robertschochia* replaces *Schochia* which was preoccupied [21]) based on a fragmentary holotype that remains the only known specimen: a set of associated upper cheek teeth (NMMNH P-9000) (Figure 13). The teeth of this specimen were relatively unworn compared to the poorly known dentition of *Wortmania* that was available for comparison at the time, but were considered to be distinct from *Wortmania* based in part on comparisons with Schoch’s [2] reconstruction of the upper dentition of *Wortmania* (Lucas and Williamson [6]: Figure 2). The specimen includes a left P4 that was not mentioned in the original description [6] and is illustrated here for the first time. Lucas and Williamson [6] distinguished *Robertschochia* from *Wortmania* by the former’s more transversely wide molars (p. 175: “wide linguolabially but narrow mesiodistally”) and possession of large molar parastyles, which were considered to be absent in *Wortmania*.

Because Schoch’s [2] reconstruction of the *Wortmania* dentition was critical to Lucas and Williamson’s [6] argument that *Robertschochia* is distinct from *Wortmania*, it is important to comment on Schoch’s descriptions based on comparative data from the remarkably complete new specimen of *Wortmania* (NMMNH P-64001). Schoch [2] provided a reconstruction of the upper molar dentition of *Wortmania* (Schoch [2]: fig. 11) illustrating relatively unworn and complete I1, C, P2-M1. Schoch [2] based this reconstruction on the type (AMNH 3394) and on four additional referred specimens that include upper teeth (see Schoch [2]: plate 17, figs. 4-7). These are: AMNH 16342, a maxilla fragment with an incomplete M2; USNM 17655, a “left P3(?)”; USNM 17654, a “right P4(?)”; and USNM 15429, a left partial maxilla with “P4-M1(?)”. The “(?)”, which were noted in Schoch’s descriptions, indicated uncertainty regarding the tooth locus.

The new specimen of *Wortmania*, NMMNH P-64001, clearly reveals the morphology of the upper first and second molars and shows that they differ significantly from that described and illustrated by Schoch [2]. The teeth that Schoch [2] tentatively identified as P4 and M1 of *Wortmania* and which formed the basis of his reconstruction of the P4 and M1 were probably misidentified, and probably represent either different tooth loci of *Wortmania*, or an unidentified taxon. This new information on the dentition of *Wortmania* gleaned from NMMNH P-64001 and the clarification of uncertainties in Schoch’s [2] upper dentition reconstruction allow for a more accurate comparison of *Wortmania* and *Robertschochia*. The upper dentition of NMMNH P-64001 closely resembles that of the holotype of *Robertschochiasullivani* (NMMNH P-9000; Figure 13) and we therefore find the latter to be a junior subjective synonym of *Wortmaniaotariidens* .

The upper molars of the new *Wortmania* specimen (P-64001) (Figure 8D-G) are closely similar to those of the *Robertschochia* holotype (P-9000), but show more occlusal wear. They are similar in their overall proportions, including transverse width (Table 1), differ little in size, and exhibit similar development and placement of the parastyle. The upper molars of P-64001 and P-9000 show similar development of an ectocingulum. P-64001 has a complete, or nearly complete, cuspate cingulum on M1 and a rounded shoulder on M2 (Figure 8J). The upper molars of P-9000 are damaged or partly obscured in this area, but a cuspidate ectocingulum is present for M1 (Figure 8C) and a similarly rounded shoulder on the buccal face of M2 is also present (Figure 13D). The M1 of P-9000 preserves evidence for a paraconule and distinct descending precingulum on the mesial face of the tooth basal to the paraconule area (Figure 13A-C). The presence of conules cannot be ascertained for P-64001 because of the more extensive attritional wear on the occlusal surface. However, there is no similar precingulum on the mesial face of the M1 of that specimen (Figure 8I-J). The mesial and distal surfaces of the upper molars of P-64001 (Figure 8K) also possess weak apicobasal folds similar to those preserved in the upper molars of P-9000.

NMMNH P-64001 differs in some minor details from the holotype of *Robertschochia* that we consider to most likely represent individual variation. For example, the P3 of both specimens appear to be closely similar in size and morphology, but the P4 of P-64001 (Figure 8I-J) differs from that of P-9000 (Figure 13A, C) in possessing a distinct parastyle rather than a low bulge in the parastyle position.

We are unable to reconcile the new specimen from New Mexico and the holotype of “*Robertschochia*” with the specimen that Schoch [2] referred to *Wortmaniaotariidens* , USNM 15429, and tentatively identified as P4-M1. We believe that Patterson [12] incorporated information from USNM 15429 in his composite drawing of the upper dentition of *Wortmania* ( [12]: fig. 1a). He evidently interpreted these teeth to represent M1-2. The anterior of the two teeth shows considerably more occlusal wear than the posterior, which is consistent with Patterson’s interpretation, but not Schoch’s, because the P4 typically erupts after the M1 in early placental mammals [34–36] and this pattern is observed in other taeniodont taxa (e.g., *Conoryctes*; personal observation). Both teeth of USNM 15429 are similar in length to P4 and/or M1 or M1-2 of P-64001 and P-9000 (Table 1), although the “P4” length in USNM 15429 is about 15 percent greater than P4 length and 4 percent greater than M1 length of NMMNH P-64001, which is larger than P-9000. Both “P4” and “M1” of USNM 15429 are mesiolingually narrower than the corresponding teeth (both P4-M1 or M1-2) of P-64001 and P-9000. The “P4” is highly worn and all occlusal details have been obliterated, but the “M1” preserves a distinct metacone and remnants of conules, especially a distinct metaconule, and unlike the M1s of NMMNH P-64001 and P-9000, lacks a distinct parastyle or a low bulge in the parastylar area. Based on this, we suggest that the teeth of USNM 15429 likely represent P3-4. The interpretation of the distal tooth as P4 makes the tooth similar to the P4 of P-64001 and P-9000 in terms of relative length and width (length/width = 0.69 for USNM 15429 versus 0.64 for P-64001 and 0.58 for P-9000) and morphology.

With that being said, there are some marked differences between the two teeth of USNM 15429 and P3-P4 in both the new specimen of *Wortmania* (P-64001) and the holotype of “*Robertschochia*” (P-9000). For example, in USNM 15429 the distal tooth is significantly larger than the P4 of P-64001 (13 percent larger). Like the P4 of P-9000 (Fig. 13A, C), the distal tooth of USNM 15429 lacks a distinct parastyle, but the tooth differs from the P4 of both P-9000 and P-64001 (Fig. 8G-E) in apparently possessing a distinct metacone and more developed conules. Unfortunately, the paucity of specimens certainly referable to *Wortmania* makes it difficult to assess individual variation. The rarity of well-preserved taeniodont dentitions also makes it difficult to confirm whether the teeth of USNM 15429 really do represent P3-4. In any case, USNM 15429 differs so substantially from both P-64001 and P-9000, regardless of whether the teeth are P3-4, P4-M1, or M1-2, that we suggest that it is not referable to *W* *. otariidens* . We note, however, that this conclusion might change with the discovery of additional specimens that would indicate that there is significant variation in P4 morphology among individuals of *Wortmania*. We also note that we are currently unable to offer a compelling alternative taxonomic identification USNM 15429 at this time.

### New Information on the Braincase and Postcranium of *Wortmania*

The new specimens of *Wortmania* also offer important new information on the braincase and postcranium of this poorly known taeniodont. The partial braincase of NMMNH P-64001 provides for the first time information regarding the squamosal and a portion of the occipital region of *Wortmaniaotariidens* . The lateral and ventral portions of the braincase (Figure 5E-G) are similar to those described by Schoch [2] for *Ectoganus* and *Stylinodon* in that the glenoid fossa is large and transversely elongated, and the posterior root of the squamosal is thin and rod-like.

The partial forelimb of NMMNH P-19460 (Figures 9-12) is the first postcranial material to be referred to *Wortmaniaotariidens* outside of the type. The new specimen does not include the dentition, but some of the elements overlap with those of the type including the ulna, radius, metacarpal II, and partial ungual, and in all cases are essentially identical. Furthermore, the forelimb bones of *W* *. otariidens* and other stylinodontids are distinctive (e.g., large size, short and stout metacarpal II with distinctive distal articular surface, laterally compressed and deep unguals) and after the synonymy of *Robertschochiasullivani* with *Wortmaniaotariidens* , there is no evidence for a second Puercan stylinodontid. Therefore we are confident in referring this new specimen to *W* *. otariidens* .

This new specimen includes new information for *Wortmania* including portions of the ulna, radius, the partial magnum, and metacarpals III and IV that were hitherto unknown for the genus. These bones are all found to be very similar, but smaller, to the corresponding bones of *Psittacotherium* (AMNH 2453) as previously described by Wortman [10], Matthew [11], and Schoch [2]. However, as Schoch [2] noted, the partial manus of AMNH 2453 has subsequently been glued together and set in plaster, hampering examination.

### Functional Morphology

Stylinodontid taeniodonts are remarkable for their extreme dental and postcranial specializations, which evolved early in the Paleocene. Mature taeniodont specimens show a high degree of occlusal wear on all teeth, which often obliterates much of the original crown morphology. Koenigswald et al. [4] suggested (p. 1803) a diet of “very coarse type browse” for the later stylinodontid taeniodonts *Ectoganus* and *Stylinodon*, and proposed that the heavy dental wear may be the result of the relatively thin enamel present in the teeth of these animals coupled with their coarse diet. They postulated that these taeniodonts likely engaged in some hard object feeding, but clearly had an unusual diet with more variability than is found in many ungulates. *Wortmania* shares a similar, albeit much less derived, tooth morphology with derived stylinodontids like *Ectoganus* and *Stylinodon*, and possesses similar patterns of wear. This suggests that it had adopted a similar masticatory style and diet as these derived taxa.

Schoch [2] postulated a two-phase mastication pattern in Taeniodonta, as is found in primitive placental mammals. In his model of mastication, the mandible first moved slightly forward during phase 1, and then moved transversely so that the lower teeth ground laterally against the upper teeth. Turnbull [26] disagreed with this model for describing mastication in the derived taeniodont *Stylinodon* and instead argued for an “orthal or weakly propalinal” power stroke in that taxon. Turnbull [26] suggested that tooth morphology and the massive, fused symphysis of *Stylinodon* would have restricted transverse movements of the lower jaw. No *Wortmania* specimens, including the new mandibles described here, show evidence for fusion of the mandibular symphysis, but the symphysis is large. Furthermore, the canines of *Wortmania* are especially large. We suggest that the canines of *Wortmania* may have acted as occlusal guides, which along with the large symphysis restricted lateral movements of the mandible.

This hypothesis is based on patterns of dental wear in *Wortmania*. Koenigswald et al. [4] argued that at least for *Stylinodon*, a one-phase power stroke directed more or less orthally was the most likely masticatory stroke. The wear on the upper molars of *Wortmania* supports a more or less orthal, one-phase power stroke during mastication for *Wortmania* as well. This is because occlusal wear of the upper molars is largely restricted to the lingual surface, rather than occurring over the entire occlusal surface (Figure 8I-J). Wear facets on the premolars of *Wortmania* are largely restricted to the apices of the major cusps and to transversely narrow bands on the mesial and distal faces of the paracones/protoconids (Figures 7L, O-P, and 8G-E), suggesting that upper and lower teeth articulated in an alternating pattern between upper and lower teeth as in *Stylinodon* [4,26]. Also, the upper molars of *Wortmania* each developed a saddle-shaped wear facet (Figure 8I, K) as is seen in derived stylinodontids (e.g., *Ectoganus* and *Stylinodon*), which are mesiodistally convex and lingobuccally concave (e.g., [4,26]) However, unlike *Stylinodon*, the cheek teeth of *Wortmania* were essentially tightly packed (Figures 7H, 8E-K).

The wear pattern of *Wortmania* and more derived stylinodontids appears to have been different from the that in the putative basal stylinodontid *Schowalteria*, in which the occlusal surface is worn nearly completely flat and the wear facet completely encompasses the paracone and metacone, leaving only an outline of the buccal side of the bases of these cusps remaining [7]. This suggests that mastication in *Schowalteria* included a transverse grinding phase rather than a more limited, mostly orthal power stroke.

The *Schowalteria* condition may be unique among taenidonts, however. Among other non-stylinodontid taeniodonts, *Onychodectes*, *Conoryctes*, and possibly some other taxa also appear to be similar to *Wortmania* in exhibiting occlusal tooth wear indicative of limited transverse movement during mastication. The upper cheek teeth of *Onychodectes* and *Conoryctes*, taxa for which numerous specimens are available, often show wear that is most prominent on the protocone and lingual faces of the para- and metacones, with relatively less wear on the buccal faces of the latter cusps and ectocingulum. Moreover, specimens of *Conoryctes* (e.g., NMMNH P-19976) often show very distinctive wear facets on cheek teeth that are comprised of deep concavities that are restricted to a small portion of the entire occlusal surface of each cheek tooth, and which are adjacent to relatively unworn portions of the occlusal surfaces, such as the tooth margin and/or parts of the lower molar trigonids. This sort of occlusal wear could only have been formed with a mostly orthal power stroke.

Several taeniodonts possess postcranial adaptations that are typical of animals that are specialized for scratch-digging (e.g., [2,26,37]). Such mammals typically show forelimb adaptations for increasing power and control of the forefoot digits as the animal drives its claws into the substrate, powerfully flexes its digits, and draws its forefeet back toward the body [38,39]. Scratch-digging requires alternate flexion and extension of the forelimb, as well as pronation and supination [37]. Postcranial adaptations for scratch-digging are often seen in the forelimb and include relatively short distal forelimb elements, relatively large distances between muscle origins/insertions to increase moment arms, relatively large muscle masses, and various characters developed to strengthen joints to prevent hyperextension of phalanges and prevent lateral dislocation of digits [37,40,41]. Osteological correlates typically developed in living scratch-diggers include a relatively short forearm and manus, with short and wide metacarpals and proximal and medial phalanges, a long olecranon process of the ulna, large muscle attachments on the scapula and humerus for powering the upper arm as well as for the origin of forearm pronators and supinators and manual flexors and extensors, squared articular surfaces or bony stops, and deep splines and grooves of the metacarpal and phalangeal joints [37,40,41]. Some scratch-diggers have long, narrow, and sharp claws to cut hard soil, but others have broader claws and a wide manus for digging in soft substrates [40,41].

All Paleogene stylinodontid taeniodonts are represented by at least parts of their postcranial skeletons. These possess specializations that are consistent with scratch-digging. These are well-developed in *Psittacotherium* and later taeniodonts [2] which are characterized by massive bones of the forelimbs; a long and well-developed deltopectoral crest on the humerus; large entepicondyle and large supinator crests of the humerus; a long olecranon process of the ulna; relatively short forearms bones; short metacarpals, proximal, and intermediate phalanges; and large laterally compressed unguals. The forearm skeleton of *Wortmania* was hitherto relatively more poorly known than those of later stylinodontids such as *Psittacotherium* and *Stylinodon*. A single, large, deep, and laterally compressed manual ungual and a short and stout second metacarpal preserved with the type of *Wortmania* (AMNH 3324; Figure 3) indicated that it possessed at least some specializations for scratch-digging and that stylinodontids had evolved at least some of these relatively extreme specializations early in the Paleocene. The new specimen, NMMNH P-64001, provides new information that further confirms forearm adaptations for scratch-digging in *Wortmania*.

In the new specimen, the radius and ulna (Figures 9-10) are robust. The length of the humerus is unknown, but based on the size of the semilunar notch of the ulna, the distal condyle of the humerus was large. The interosseus ligaments between the ulna and radius were well-developed and the placement, shape, and orientation for the articular surfaces between the radius and ulna suggests that the forelimb was habitually pronated with restricted rotation of the radius. Perhaps surprisingly, supination may have been restricted. This is contrary to what is seen in most scratch-diggers which, according to Hildebrand [40,41] and Coombs [37], typically pronate and supinate their forelimbs. The inference that *Wortmania* was incapable of a large degree of supination is based on the description of Rose ( [42]: 367), who stated that a nearly flat ulnar facet on the radius (the radial notch) like that of *Wortmania* allows little supination and is often associated with terrestrial and possibly digging behavior. The radial notch is nearly flat and faces more dorsally in *Wortmania* than in *Onychodectes*. Later stylinodontids had more dorsally facing notches than in *Wortmania*. The distal end of the radius of the new *Wortmania* specimen is large and smoothly flat to concave (not subdivided into separate lunar and scaphoid facets), but does not have the large dorsal flanges present in *Psittacotherium* (NMMNH P-47687), to channel the extensor tendons. Also, the distal end of the ulna has a relatively small styloid process compared to *Psittacotherium* (NMMNH P-47687) and later stylinodontids [2].

As in other scratch diggers, *Wortmania* possessed distinct musculoskeletal modifications that provided increased strength in flexing the larger digits. *Wortmania* possesses short and stout metacarpals with enlarged tuberosities on the proximodorsal surfaces of metacarpals II and III (Figures 11-12) which are found in many fossorial mammals [41,42]. The unguals also have a large flexor process on the ventral surface (Figure 11H-I) that provided an increased attachment area for, and enhanced the power of, the deep digital flexors [41,42].

Furthermore, as in many scratch diggers, *Wortmania* has features of the manus that strengthened joints against dislocation and hyperextension (e.g., [41]: Figure 6-3). The joints between the metacarpals and proximal phalanges are relatively flat, making the joint rigid as in many plantigrade diggers. These features are also seen in *Manis*, the pangolin, as well as the leptictid *Prodiacodon* (NMMNH P-60135) and the putative basal palaeanodont *Escavadodon* ( [30]: Figure 10), and all other taeniodonts for which the distal metacarpals are known (e.g., *Onychodectes, Conoryctes, Psittacotherium, Ectoganus*, and *Stylinodon*; below). These joints are further strengthened by a large median keel flanked by grooves that is restricted to the plantar half of the joint. The proximal articular surface of the ungual phalanx is deep and has a long radius of curvature. The ungual phalanx (best seen in the complete ungual of AMNH 3394, Figure 3D) has a large flexor tubercle that, in addition to providing an increased attachment area for the digital flexors, also formed a bony stop that limited extreme digit flexion and thus dislocation.

*Onychodectes* is the most basal taeniodont for which postcrania are known [2,3]. Schoch [2] and Lucas et al. [3] concluded that *Onychodectes* and other “conoryctids” (e.g., *Conoryctella*, *Huerfanodon*, *Conoryctes*) possessed generalized postcranial skeletons with few specializations. The unguals of the manus and pes were described as being relatively small, although Lucas et al. [3] suggested that the (p. 265) “relatively heavy forelimbs and claws” may have allowed conoryctids to harvest subsurface food items. We find that *Onychodectes*, considered by some to be a basal taeniodont (e.g., [2,3]), possesses several key features indicative of scratch-digging. The humerus has a relatively long deltopectoral crest that extends about half the length of the bone for attachment of large and well-developed deltoid and pectoralis muscles for powering the upper arm, a broad distal end with a large supinator crest, and entepicondyle for well-developed supinator and manual flexors and extensors. The ulna has a relatively long olecranon process that curves medially, providing a long in-lever for the triceps and increased areas for origin of the digital and carpal flexors, and also increased the effective force of the pronator [40,41]. The dorsoproximal surfaces of metacarpals II and III bear prominent extensor tubercles, and the distal end of the metacarpals is flat over the planter half of the joint, making the joint rigid, and the manual ungual is enlarged and laterally compressed. These features of a manual ungual with AMNH 16528 were not noted by Schoch [2] and his drawings seem to portray a small and non-compressed ungual, although original specimens show that the ungual is indeed large and laterally compressed. These adaptations are similar to what is found in some leptictids [42], but not as extreme as those seen in the putative basal palaeanodont *Escavadodon* [30].

Other “conoryctids”, including *Conoryctella*, *Huerfanodon*, and *Conoryctes*, were also described as having “primitive” and “generalized” postcranial skeletons [2,3]. However, little of the postcranial skeleton has been described for these taxa. A partial ulna is associated with the holotype of *Conoryctellapattersoni* (NMMNH P-25056) and a partial radius, a partial humerus, and a fragment of a shaft of a tibia are associated with the type of *Hexodonmolestes* (AMNH 3396), which is regarded as a junior synonym of *Conoryctes* *comma* [2]. Schoch [2] also suggested that a partial left manus (USNM 23483) may be referable to *Conoryctes* based on its larger size, but this manus bears close resemblance to the manus of *Onychodectes* (based on AMNH 16528). Schoch [2] concluded that based on these specimens, *Conoryctes* lacked any specialized postcranial adaptations. New specimens, however, are clearly needed to assess whether Schoch was correct, or alternatively, whether *Huerfanodon* and *Conoryctes* may also have digging adaptations like those in the supposedly “generalized” *Onychodectes* that we identify here.

*Wortmania* possesses all the digging adaptations found in *Onychodectes* (and perhaps other “conoryctid” taeniodonts, based on limited postcranial material), and as in all stylinodontid taeniodonts, it is notably larger and more robust than “conoryctid” taxa. The younger stylinodontids—the early to late Paleocene *Psittacotherium*, early Eocene *Ectoganus*, and middle to late Eocene *Stylinodon*—are progressively larger and have more robust postcranial skeletons than *Wortmania*. The more robust forelimbs in the younger stylinodontids, culminating in *Stylinodon*, likely made them stronger and more effective diggers. In addition, *Psittacotherium*, *Ectoganus*, and *Stylinodon* possess larger and a more distally projecting styloid process of the ulna than *Wortmania*, and this probably facilitated more powerful, and better controlled positioning of the manus through the entire range of rotation of the forelimb from pronation to supination. The manus of *Stylinodon* is shorter compared to the radius than that of *Wortmania* (metacarpal III length/radius length = 0.32 for *Wortmania* [see Table 4] and 0.27 for *Stylinodon* based on measurements in Turnbull [26]) *Stylinodon* appears to have had a carpus that was probably stronger and more stable, with forces being transmitted more directly from metacarpal III through the magnum than in *Wortmania* and probably also *Psittacotherium*. The manus of *Stylinodon* is supplemented by a number of large sesamoid bones that served and protected the flexor tendons [26]. No sesamoid bones have been recovered for *Wortmania* or for any other taeniodont, suggesting that they all had relatively smaller and less powerful flexor muscles and tendons than *Stylinodon*.

In summary, all taeniodonts for which we have adequate postcranial skeletons to assess functional adaptations of the forelimb possess at least some indicators for scratch-digging. *Wortmania* was larger and more robust than *Onychodectes* and “conoryctid” taeniodonts (*Conoryctes* and *Huerfanodon*) and was therefore a more powerful digger. *Wortmania*, in turn, was smaller and had a less robust postcranial skeleton than later stylinodontid taeniodonts such as *Psittacotherium* and *Stylinodon*.

### Phylogenetics

The phylogeny of taeniodonts has only been examined in cursory detail. Schoch [2] presented a hypothesis of taeniodont relationships based on the distribution of different characters, but this phylogeny was drawn by hand and did not result from a numerical cladistic analysis. Lucas and Williamson [6] and Lucas et al. [3] discussed taeniodont phylogeny based on a limited number of dental characters, including new morphological information from what they considered to be a new early Paleocene taxon, *Robertschochia*, but once again this was not based on a computer-generated cladistic analysis. Most recently Rook and Hunter [8] published a genealogy of taeniodonts based on a numerical cladistic analysis of dental characters that were largely developed based on the descriptions of Schoch [2], as well as specimens examined by the authors.

We are currently undertaking a reanalysis of taeniodont phylogeny that will incorporate a full suite of dental, cranial, and postcranial characters. This analysis will include information from several new taeniodont specimens from New Mexico, including the new material of *Wortmania* described here, as well as a comprehensive review of historic specimens in museum collections. This analysis will be presented elsewhere, and must follow the description of other new taeniodont specimens from New Mexico. In the meantime, in this paper we discuss the phylogenetic character scores of *Wortmania* in the dental matrix of Rook and Hunter [8], revise these scores based on information from the new specimens described here, comment on other pertinent character scores for various taxa (including those for “*Robertschochia*”), and present the results of a cladistic analysis using a revised version of the Rook and Hunter [8] matrix.

Rook and Hunter [8] scored both *Robertshochia* (as *Schochia*) and *Wortmania* as separate taxa in their analysis. *Robertschochia* was not scored for many characters due to missing data (?), as its holotype specimen is highly fragmentary and incomplete. *Robertschochia* and *Wortmania* were scored differently for six characters. In the following list, the character number is given first, followed by the scores for *Robertschochia* and *Wortmania*, respectively, in parentheses: 11 (2, 0), 12 (0, 1), 14 (0, 1), 28 (1, 0), 29 (0, -; note the scoring “-” for *Wortmania* mistakenly appears twice), 34 (1,2). These characters and the relevant scores for “*Robertschochia*” and *Wortmania* are here discussed individually. In each case, our final character score for *Wortmania* is for a single operational taxonomic unit that is based on all known specimens of *Wortmania* and the “*Robertschochia*” holotype, following our synonymization of these two taxa:

Character 11: Rook and Hunter [8] scored the P4 of *Wortmania* as “triangular” (0) and *Robertshochia* as “molariform” (2). The P4 of “*Robertschochia*” (based on NMMNH P-9000) and the new *Wortmania* specimen NMMNH P-64001 are similar in lacking evidence of a metacone and conules, although it is possible that wear has obliterated evidence of their presence. Based on this, we tentatively score *Wortmania* as possessing a submolariform P4 (1) for character 11.
Character 12: Rook and Hunter [8] scored *Wortmania* as having an “incipient” (1) P4 parastyle and “*Robertschochia*” (based on NMMNH P-9000) as lacking a parastyle (0), but a P4 parastyle is present in the new *Wortmania* specimen NMMNH P-64001. We therefore score *Wortmania* as being polymorphic (0/1) for a P4 parastyle.
Character 14: A P4 metastyle is present in both “*Robertschochia*” (NMMNH P-9000) and the new *Wortmania* specimen P-64001 and we have scored this as “small” (2) for *Wortmania* rather than as “absent” (0) or “incipient” (1) as scored for “*Schochia*” and *Wortmania*, respectively, for character 14 by Rook and Hunter [8].
Character 28: The upper molars of “*Robertschochia*” (NMMNH P-9000) and the new *Wortmania* specimen P-64001 both are heavily worn, but show clear remnants of conules. We therefore score *Wortmania* as possessing “small” (0) conules following Rook and Hunter’s [8] scoring for “*Schochia*” for this character.
Character 29: The upper molar conules of “*Robertschochia*” (NMMNH P-9000) and the new *Wortmania* specimen P-64001 appear to have been positioned approximately midway between the para- and metacones and the protocone and therefore are scored as being “labial” (state 0) following Rook and Hunter’s scoring for “*Schochia*” for this character.
Character 34: We find that the M2 of *Wortmania* (based on NMMNH P-9000 and P-64001) is “subequal in size with, or slightly smaller than, M1” (2) rather than “shorter but more transverse than M1” (1), following Rook and Hunter’s [8] scoring for “*Schochia*” for this character (see Table 1).

Following from the above discussion, we ran a revised version of the Rook and Hunter [8] matrix. “*Robertschochia*” (“*Schochia*”) was removed from the analysis because we consider this taxon to be a subjective junior synonym of *Wortmania* (see above). We incorporated the above amendments to character scores: the creation of a single *Wortmania* OTU subsuming “*Robertschochia*” and updates to some *Wortmania* character scores based on the new material described in this paper. Our set of revised character scores for *Wortmania* is provided in Table 5. No other changes to the Rook and Hunter dataset were made.

**Table 5:** Revised scoring for *Wortmaniaotariidens* as discussed in text.

2221100200	10/1120100?1	1110120002	0212111

We subjected our dataset to a parsimony analysis in TNT v. 1.1 [43]. As an initial step, we analyzed the matrix under the ‘New Technology search’ option, using sectorial search, ratchet, tree drift, and tree fuse options with default parameters. The minimum length tree was found in 10 replicates, which aimed to sample as many tree islands as possible. The recovered trees were then analyzed under traditional TBR branch swapping, to more extensively explore each tree island.

Running the analysis with all characters unordered (which was not done by Rook and Hunter, but which was done here to gauge the effects of character ordering on the topology) resulted in six most parsimonious trees of 105 steps (Figure 14A; Consistency Index = 0.648, Retention Index = 0.667). The strict consensus of these trees recovers a monophyletic Taeniodonta that is in a polytomy with both species of *Procerberus*. *Alveugena*, *Schowalteria* and *Onychodectes* form successive sister taxa to a clade with a basal polytomy consisting of *Conoryctella, Conoryctes*, *Huerfanodon*, and a clade of more derived taeniodonts (Stylinodontidae). This derived clade includes *Wortmania* and*Psittacotherium* as successive outgroups toa clade of *Ectoganus* plus *Stylinodon*. The analysis was run with some multistate characters ordered following Rook and Hunter [8; characters 1, 2, 3, 4, 6, 7, 8, 10, 11, 12, 13, 14, 15, 21, 23, 24, 25, 26, 27, 30, 32, 34, 36]. Character 31 was also considered an ordered character following the recommendation of Hunter (personal communication) who suggested that an asterisk indicating that character 31 was an additive (ordered) character was mistakenly omitted in Rook and Hunter [8]. This resulted in one most parsimonious trees with a length of 121 steps (Figure 14B; Consistency Index = 0.562, Retention Index = 0.679). The tree shows a monophyletic Taeniodonta that is sister taxon to a clade of both species of *Procerberus*. *Alveugena*, *Schowalteria* and *Onychodectes* form successive sister taxa to a clade consisting of a monophyletic Conoryctidae (consisting of *Conoryctella*, *Conoryctes*, *Huerfanodon*) and its sister taxon, a monophyletic Stylinodontidae in which *Wortmania* and *Psittacotherium* form successive sister taxa to a sister-taxon pair of *Ectoganus* plus *Stylinodon*. Characters supporting the individual nodes are presented in Appendices S3 and S4.

The results of our analyses differ from the phylogeny obtained by Rook and Hunter ( [8]: Figure 1) in several respects. First, *Onychodectes* is placed in a more basal position, basal to all conoryctid and stylinodontid taeniodonts whereas Rook and Hunter [8] found *Onychodectes* as the sister taxon to Stylinodontidae (i.e., nested between Conoryctidae and Stylinodontidae on the phylogeny). Second, we agreed with Rook and Hunter [8] in recovering a monophyletic Conoryctidae, with *Conoryctella* as the most basal member, but only in the analysis with ordered characters. In the analysis with unordered characters *Conoryctella*, *Huerfanodon*, and *Conoryctes* fall into a polytomy that is sister to Stylonodontidae. Third, only in the analysis with ordered characters is *Wortmania* placed definitively as the most basal derived taeniodont (stylinodontid), as in the analysis with unordered characters *Wortmania* and *Psittacotherium* fall into a basal stylinodontid polytomy. In the unordered analysis there remains considerable phylogenetic uncertainty because of the polytomies, and only future analyses of more characters and taxa, including especially several taxa that are best represented by New Mexico specimens, can solve this vexing issue.

## Conclusion

**Figure 15:**
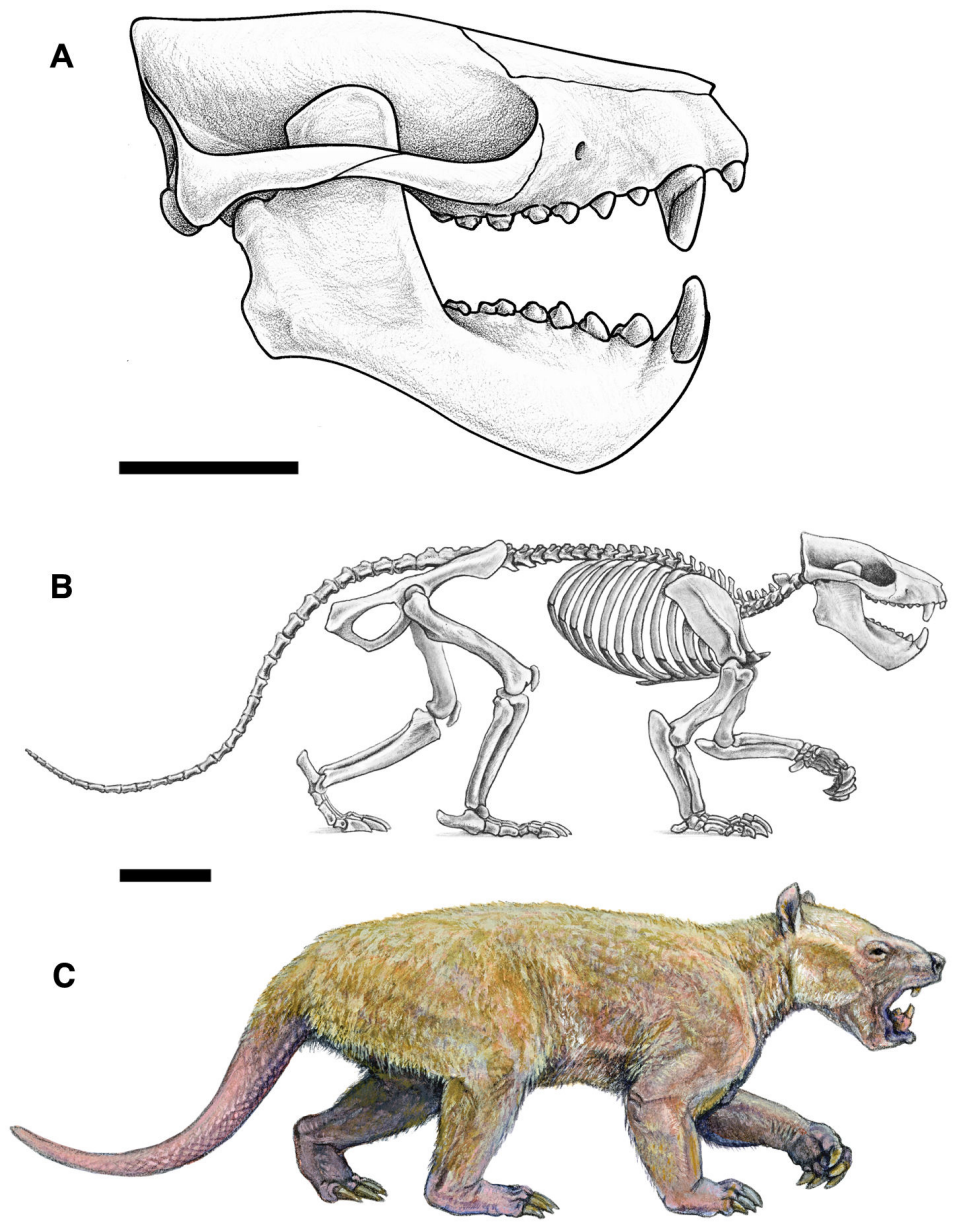
Reconstruction of *Wortmaniaotariidens* . Artwork by Matt Celeskey, 2012.

Three new specimens of the rare early Paleocene taeniodont *Wortmaniaotariidens* are described here. These include one of the most complete cranial specimens known for the species, which is the first *Wortmania* specimen to include associated upper and lower teeth, and another fossil that is only the second known specimen that includes postcranial remains. These specimens provide new anatomical information on this rare genus, which was one of the first mammals to evolve moderately large size and extreme functional adaptations (for scratch-digging and a high-wear diet) after the extinction of the dinosaurs (Figure 15). This new information allows for comparison with other, more fragmentary taeniodont specimens and leads us to conclude that *Robertschochiasullivani* is a junior synonym of *Wortmaniaotariidens* . These specimens also provide new data with which to examine taeniodont phylogeny and functional morphology. A revised version of a published phylogenetic analyses of Taeniodonta, which incorporates new morphological data from the new specimens, suggests that “Conoryctidae”, a previously proposed clade of small, taeniodonts with supposedly “generalized” skeletons, is monophyletic, but this clade is only found with ordered characters and falls apart when unordered characters are used. *Wortmania*, is placed within the subclade Stylinodontidae, a group of larger-bodied and more robust taeniodonts, as previously proposed. Preliminary analysis also suggests that all taeniodonts for which postcrania are known possess at least some postcranial adaptations for scratch-digging, and that *Wortmania* was somewhat intermediate in its digging capabilities between more generalized and gracile taxa such as *Onychodectes* and the largest, most robust stylinodontids such as *Stylinodon* and *Psittacotherium*.

## Supporting Information

Appendix S1 **Taxon-character matrix with characters unordered.**
(TNT)

Appendix S2 **Taxon-character matrix with characters ordered.**
(TNT)

Appendix S3 **Characters in common on the most parsimonious trees diagnosing the selected nodes on the strict consensus tree resulting from the analysis run with characters unordered (Figure 14A).**
(DOCX)

Appendix S4 **Characters in common on the most parsimonious trees diagnosing the selected nodes on the strict consensus tree resulting from the analysis run with characters ordered (Figure 14B).**
(DOCX)
