# Supplementary material for: New Specimens of the Rare Taeniodont Wortmania (Mammalia: Eutheria) from the San Juan Basin of New Mexico and Comments on the Phylogeny and Functional Morphology of “Archaic” Mammals
Source: PLoS One. 2013 Sep 30;8(9):e75886. doi: 10.1371/journal.pone.0075886 (PMC3786969; doi:10.1371/journal.pone.0075886)
Supplement: Appendix S3 — Characters in common on the most parsimonious trees diagnosing the selected nodes on the strict consensus tree resulting from the analysis run with characters unordered (Figure 14A). (DOCX) [file pone.0075886.s003.docx]

Node A:

All trees:

No synapomorphies

Node B:

All trees:

Char. 12: 3 --> 2

Char. 14: 3 --> 2

Char. 26: 0 --> 1

Char. 27: 0 --> 1

Char. 35: 0 --> 1

Node C:

All trees:

Char. 22: 0 --> 1

Char. 23: 0 --> 1

Char. 33: 0 --> 1

Some trees:

Char. 15: 0 --> 1

Node D:

All trees:

Char. 11: 0 --> 1

Char. 30: 1 --> 2

Char. 32: 0 --> 2

Char. 34: 0 --> 2

Node E:

All trees:

Char. 7: 0 --> 1

Char. 26: 1 --> 2

Char. 27: 1 --> 2

Char. 28: 1 --> 2

Some trees:

Char. 4: 0 --> 1

Char. 25: 0 --> 2

Node F:

All trees:

Char. 5: 0 --> 1

Char. 16: 0 --> 1

Some trees:

Char. 1: 0 --> 2

Char. 2: 01 --> 2

Char. 12: 2 --> 0

Char. 13: 3 --> 1

Char. 15: 1 --> 2

Node G:

All trees:

Char. 7: 1 --> 2

Some trees:

Char. 4: 1 --> 3

Char. 6: 0 --> 2

Char. 10: 1 --> 2

Char. 11: 1 --> 2

Char. 24: 0 --> 2
