# Supplementary material for: New Specimens of the Rare Taeniodont Wortmania (Mammalia: Eutheria) from the San Juan Basin of New Mexico and Comments on the Phylogeny and Functional Morphology of “Archaic” Mammals
Source: PLoS One. 2013 Sep 30;8(9):e75886. doi: 10.1371/journal.pone.0075886 (PMC3786969; doi:10.1371/journal.pone.0075886)
Supplement: Appendix S4 — Characters in common on the most parsimonious trees diagnosing the selected nodes on the strict consensus tree resulting from the analysis run with characters ordered (Figure 14B). (DOCX) [file pone.0075886.s004.docx]

Node A :

All trees:

No synapomorphies

Node B :

All trees:

Char. 9: 0 --> 2

Char. 10: 0 --> 2

Node C :

All trees:

Char. 11: 3 --> 2

Char. 13: 3 --> 2

Char. 25: 0 --> 1

Char. 26: 0 --> 1

Char. 34: 0 --> 1

Node D :

All trees:

Char. 14: 0 --> 1

Char. 21: 0 --> 1

Char. 22: 0 --> 1

Char. 32: 0 --> 1

Node E :

All trees:

Char. 10: 0 --> 1

Char. 20: 0 --> 1

Char. 29: 1 --> 2

Char. 31: 1 --> 2

Char. 33: 1 --> 2

Node F :

All trees:

Char. 3: 0 --> 1

Char. 24: 0 --> 12

Char. 25: 1 --> 2

Char. 27: 1 --> 2

Node G :

All trees:

Char. 26: 1 --> 2

Char. 30: 0 --> 1

Node H :

All trees:

Char. 8: 0 --> 1

Char. 9: 0 --> 1

Char. 30: 1 --> 2

Node I :

All trees:

Char. 0: 1 --> 2

Char. 1: 1 --> 2

Char. 4: 0 --> 1

Char. 7: 1 --> 2

Char. 12: 23 --> 1

Char. 15: 0 --> 1

Node J :

All trees:

Char. 3: 1 --> 2

Char. 5: 0 --> 1

Char. 9: 0 --> 1

Char. 13: 2 --> 0

Char. 14: 1 --> 2

Char. 20: 1 --> 0

Char. 28: 0 --> 1

Node K :

All trees:

Char. 3: 2 --> 3

Char. 5: 1 --> 2

Char. 6: 1 --> 2

Char. 9: 2 --> 0

Char. 10: 1 --> 2

Char. 23: 1 --> 0
